# Design and methodology for a proof of mechanism study of individualized neuronavigated continuous Theta burst stimulation for auditory processing in adolescents with autism spectrum disorder

**DOI:** 10.3389/fpsyt.2024.1304528

**Published:** 2024-02-08

**Authors:** Lindsay M. Oberman, Sunday M. Francis, Lysianne Beynel, Megan Hynd, Miguel Jaime, Pei L. Robins, Zhi-De Deng, Jeff Stout, Jan Willem van der Veen, Sarah H. Lisanby

**Affiliations:** ^1^ Noninvasive Neuromodulation Unit, Experimental Therapeutics and Pathophysiology Branch, National Institute of Mental Health, National Institutes of Health, Bethesda, MD, United States; ^2^ Clinical Affective Neuroscience Laboratory, Department of Psychology & Neuroscience, University of North Carolina, Chapel Hill, NC, United States; ^3^ Magnetoencephalography Core, National Institute of Mental Health, National Institutes of Health, Bethesda, MD, United States; ^4^ Magnetic Resonance Spectroscopy Core, National Institute of Mental Health, National Institutes of Health, Bethesda, MD, United States

**Keywords:** continuous theta burst stimulation, Autism spectrum disorder, magnetoencephalography, auditory processing, magnetic resonance spectroscopy, magnetic resonance imaging, transcranial magnetic stimulation

## Abstract

It has been suggested that aberrant excitation/inhibition (E/I) balance and dysfunctional structure and function of relevant brain networks may underlie the symptoms of autism spectrum disorder (ASD). However, the nomological network linking these constructs to quantifiable measures and mechanistically relating these constructs to behavioral symptoms of ASD is lacking. Herein we describe a within-subject, controlled, proof-of-mechanism study investigating the pathophysiology of auditory/language processing in adolescents with ASD. We utilize neurophysiological and neuroimaging techniques including magnetic resonance spectroscopy (MRS), diffusion-weighted imaging (DWI), functional magnetic resonance imaging (fMRI), and magnetoencephalography (MEG) metrics of language network structure and function. Additionally, we apply a single, individually targeted session of continuous theta burst stimulation (cTBS) as an experimental probe of the impact of perturbation of the system on these neurophysiological and neuroimaging outcomes. MRS, fMRI, and MEG measures are evaluated at baseline and immediately prior to and following cTBS over the posterior superior temporal cortex (pSTC), a region involved in auditory and language processing deficits in ASD. Also, behavioral measures of ASD and language processing and DWI measures of auditory/language network structures are obtained at baseline to characterize the relationship between the neuroimaging and neurophysiological measures and baseline symptom presentation. We hypothesize that local gamma-aminobutyric acid (GABA) and glutamate concentrations (measured with MRS), and structural and functional activity and network connectivity (measured with DWI and fMRI), will significantly predict MEG indices of auditory/language processing and behavioral deficits in ASD. Furthermore, a single session of cTBS over left pSTC is hypothesized to lead to significant, acute changes in local glutamate and GABA concentration, functional activity and network connectivity, and MEG indices of auditory/language processing. We have completed the pilot phase of the study (n=20 Healthy Volunteer adults) and have begun enrollment for the main phase with adolescents with ASD (n=86; age 14-17). If successful, this study will establish a nomological network linking local E/I balance measures to functional and structural connectivity within relevant brain networks, ultimately connecting them to ASD symptoms. Furthermore, this study will inform future therapeutic trials using cTBS to treat the symptoms of ASD.

## 1 Introduction

Autism Spectrum Disorder (ASD) is a behaviorally defined complex neurodevelopmental syndrome, characterized by persistent deficits in social communication and social interaction and the presence of restricted, repetitive patterns of behavior, interests or activities (1). Since its initial description by Kanner in 1943 (2), many researchers have attempted to identify the underlying physiological cause of this behaviorally defined disorder. The predominant broad neurophysiological theory currently posits that the ASD behavioral phenotype stems from atypical brain development resulting in aberrant local and long-range connectivity within and between multiple brain regions and functional networks. However, these broad constructs of “brain development” and “connectivity”, require a nuanced approach to be applied in clinical translational research. Defining the nomological network whereby measurable outcomes within a given individual that represent these constructs and can be quantitatively probed and related to each other in a model that brings us from neurophysiological etiology to behavior has been the focus of a large number of studies (see (3, 4) for how such models have been applied to describe the ASD phenotype).

Benefitting from technological advances in Genetics, Neuroimaging, and Neurophysiological tools, several theories have emerged proposing specific mechanisms that may underlie the behavioral disorder of ASD. One such theory, first proposed by Rubenstein and Merzenich (5), implicates “Excitation/Inhibition imbalance”. On a molecular level, basic research studies suggest that brain development is dynamically and powerfully controlled by gamma-aminobutyric acid (GABA)ergic inhibitory and glutamatergic excitatory mechanisms (see (6) for a review). In their initial report, Rubenstein and Merzenich suggest that an imbalance in GABA and glutamate during early neurodevelopment may lead to functional networks whose responses are “noisier”/less reliable and undifferentiated. Based on data from human and animal models supporting this theory, we and others have suggested that abnormalities in the GABA (7) and glutamate (8) systems leading to altered network plasticity (9) may contribute to altered anatomical and less efficient functional connectivity across different brain networks (10, 11). This may manifest as alterations in diffusion derived parameters (12–14) and/or alterations in either resting state or task-related functional connectivity (15) that has been shown in language related networks. Additionally, altered connectivity may also be seen in reduced, delayed, or variable neurophysiological responses to stimuli (16). Though many have suggested a relationship between the ASD behavioral phenotype and “Excitation/Inhibition imbalance” and/or “Neural Noise”, the nomological network by which measures of E/I imbalance lead to measures of pathological network connectivity and functioning, and in turn how these processes relate to the behavioral phenotype of ASD has yet to be elucidated. Additionally, there remains debate regarding the direction of alterations (i.e. too much or too little connectivity or too much or too little noise) (10, 17–20) or no difference at all (21) and may be a consequence of whether the measure used relates to local or long-range networks, which network is being probed, and at what spatial or temporal scale.

Given the current state of the literature, with the broad goal to understand mechanisms that may be driving ASD symptom presentation to inform targeted interventions that modulate the proposed mechanism, it is critical to carefully choose outcome measures and networks that have been well-validated, aberrant in ASD, and related to the behavioral phenotype. Thus, the current study focuses on the posterior superior temporal cortex (pSTC). The pSTC is involved in temporal integration of visual, auditory and somatosensory cues of others’ behaviors and representation of a basic form of intentionality (22–26). In the auditory domain, this region comprises the secondary auditory cortex, and specifically a region known as “Wernicke’s area” critical to semantic and phonological aspects of language processing (27). Early descriptions by Kanner (28) highlight aspects of language and language processing as core symptoms of the disorder. Though the current DSM-5 diagnostic criteria does not explicitly list auditory or language processing as a core symptom, alterations in perceptual processing, specifically in the auditory domain have been established across multiple levels of analysis and have been related to not only the social and communication symptoms of ASD, but also the restricted and repetitive behavior domain (29) though the directionality of this relationship is still up for debate (30).

Neuroimaging data suggests that there may be altered functioning of both left and right hemisphere pSTC and related inferior frontal language regions in ASD in response to language processing tasks. Namely, Tanigawa and colleagues (31–38) found reduced activation of the left pSTC during an auditory word comprehension task in adolescents with ASD (31). Conversely, Just and colleagues reported increased activation of the left pSTC during a sentence comprehension task (38) and a lack of selective activation of the right hemisphere pSTC with increased sentence difficulty or the presence of intentionality information (36) in adults with ASD. Furthermore, Wang and colleagues found increased activation in both the left and right pSTC in a study of irony comprehension, suggesting that more effortful processing is needed to interpret the intended meaning (34). Additionally, aberrant response in the left pSTC to auditory and speech stimuli predicts language processing impairments in both ASD (32) and other developmental and neurological disorders (39, 40).

At a functional network level, the pSTC is part of a broader language processing network, connected structurally through the arcuate fasciculus, linking it to the inferior frontal gyrus (IFG, and specifically “Broca’s Area”). Disruption in connectivity of this network is thought to impact both receptive and expressive language (27). Adolescents and adults with ASD show reduced task-related functional connectivity in this network and aberrant recruitment of pSTC and IFG in response to specific language tasks (38, 41). This network can be reliably detected by both resting-state and task-related functional magnetic resonance imaging (fMRI) scans (42).

Based on both animal model (43) and human fMRI data (43) linking E/I imbalance in networks related to auditory processing and the link between dysfunction in these regions and ASD symptoms, human clinical trials of glutamatergic and GABAergic drugs in children and adults with ASD are ongoing. However, it is unclear whether modulating concentration of these neurotransmitters may improve the pathological functional network connectivity or the behavioral phenotype. Thus, consistent with the suggestion of Gonçalves and Monteiro (29), in their recent review, the current study utilizes an experimental therapeutics approach and collects measures of E/I balance (magnetic resonance spectroscopy (MRS) GABA and glutamate concentrations), network connectivity (resting state and language task-related fMRI and diffusion weighted imaging (DWI)), magnetoencephalography (MEG) measures of auditory processing to attempt to develop a nomological network linking measures of E/I balance to measures of brain network connectivity and functioning to behavior.

Though it is not feasible to directly, noninvasively measure excitation and inhibition in humans, levels of GABA (primary inhibitory neurotransmitter) and glutamate (primary excitatory neurotransmitter) concentrations can be estimated using specialized spectroscopic MRI sequences (i.e. GABA-edited MRS) (44, 45). Structural and functional network connectivity can also be assessed using specialized MRI sequences while the person is at rest or while they engage in a task (46). In the current study we will use diffusion derived parameters within the auditory radiations and the arcuate fasciculus obtained using DWI to estimate structural connectivity of these relevant tracts. Additionally, whole brain blood oxygen level dependent (BOLD) fMRI values will be obtained during rest and during a language task as a measure of functional activation in specific brain regions. The time courses of the BOLD activations in pre-defined regions of interest (ROIs) will be correlated to quantify long-range functional connectivity of language network nodes as compared to unrelated brain regions. For the physiological measure of network functioning, the current study focuses on MEG evoked fields in response to auditory stimuli (i.e., M100/M50 latency, Auditory Steady State Response (ASSR) evoked gamma response and inter-trial coherence (ITC), and Mismatch Field latency). Resting state spectral power across all the frequency bands will also be obtained. These MEG measures have been validated and reliably shown to be both aberrant in the ASD population and correlated with clinical scales assessing general ASD symptoms as well as specific language processing impairments and diffusion measures of structural connectivity (31, 47–52). These MEG outcomes were also chosen for their association with measures of E/I balance (M100/M50 (53, 54), evoked gamma power and ITC (55–57), MMF amplitude (58), and peak gamma frequency and alpha amplitude in resting state MEG (59–62)) and functional connectivity (47, 48) in ASD. Finally, it was noted that children with ASD showed greater pre-stimulus activity in these tasks, and pre-stimulus activity was related both to M100 latency and severity of language processing impairments (63), thus we will plan to evaluate this outcome as well. By obtaining these metrics in each participant, we can begin to elucidate the relationships across these levels.

Once these measures are obtained at baseline, we will then transiently modulate the system by performing a single session of noninvasive repetitive transcranial magnetic stimulation (rTMS) focused over an individually defined region of the left pSTC. Left pSTC was chosen (rather than right pSTC) for its role in language processing and robust activation in response to the auditory task, providing a target that could be reliably engaged and probed pre and post stimulation. Specifically, we will apply a continuous theta burst stimulation (cTBS) protocol. cTBS involves application of bursts of three pulses of TMS at 50 Hz, repeated at intervals of 200ms for a total of 600 pulses (200 bursts) and takes approximately 40 seconds to apply. A single session of cTBS is traditionally thought to lead to suppression of cortical excitability that lasts 20-30 minutes in a typically developing adult motor cortex (and approximately 15 minutes in a typically developing child) (64). We have shown, however, that the inhibitory effects of this same protocol last significantly longer (with an average duration of 75-90 minutes) in adults with ASD (and 30-45 minutes in children with ASD) (65).

Both glutamatergic and GABAergic neurotransmissions are thought to be involved in the mechanism of action of cTBS. Rodent and human spectroscopic imaging studies show that cTBS leads to modulation of GABAergic inhibition within the stimulated cortical regions. Specifically, rodent studies show that cTBS leads to increased cortical inhibition by reducing the expression of specific proteins (parvalbumin (PV) and calbindin D-28k (CB)) found in inhibitory interneurons. The reduced expression of these proteins in turn affected the ability of interneurons to control pyramidal cell output and dendritic integration of synaptic inputs (66). Consistent with the data from the animal literature, Stagg and colleagues (67) showed that applying a single session of cTBS to the motor cortex in healthy control human participants led to a short-term (less than an hour) increase in GABA concentration as measured by MRS compared to a non-stimulated region. Glutamate neurotransmission also appears to have a role in the observed effects of cTBS as memantine, a N-methyl-D-aspartate receptor antagonist, blocked the suppressive effects of cTBS in a double blind, placebo-controlled study (68). Additionally, in a recent study conducted by Cember and colleagues (69) cTBS led to a decrease in glutamate concentration in the targeted region of the left primary motor cortex as measured by glutamate-weighted chemical exchange saturation transfer (gluCEST) with a 7 Tesla MRI with no changes noted in a contralateral motor cortical region or in subjects receiving sham stimulation. Thus, cTBS tends to increase GABAergic and decrease glutamatergic neurotransmission, making this protocol of interest for a targeted intervention for ASD where putative pathophysiological mechanisms implicate deficient GABA and/or abnormally elevated levels of glutamate. In addition to modulating GABA and glutamate concentrations, theta burst stimulation, like other rTMS protocols, are also thought to lead to a phase reset and local entrainment of brain oscillations in the frequency band of stimulation (70, 71) suggesting there is an enhancement in the relevant physiological signal (in the case of cTBS, this would be theta oscillations). Alternatively, others have suggested that rTMS adds stochastic noise into the system (72). Though perhaps counter-intuitive, the addition of optimal levels of noise is thought to enhance the detection of sensory signals (73) and specifically in the auditory system, adding noise enhances the gamma band auditory steady state response (74, 75).

As the effects of a single session of cTBS are thought to be transient (on the order of minutes to less than 2 hours in duration), the goal of this study is to evaluate target engagement and the acute effects of cTBS on auditory/language network functioning. Thus, the cTBS session is administered within the Functional Magnetic Resonance Core Facility at the National Institutes of Health in Bethesda, Maryland, USA allowing for acquisition of imaging measures both immediately before and immediately after each cTBS session. We do not expect to induce an observable clinical/behavioral change, however, questionnaires are administered at the end of each session to assess for any observable changes in behavior or other side effects reported by participants. If successful, this study may inform future therapeutic trials aiming to treat the symptoms of ASD by modulating network functioning through pharmacological or device-based manipulations of glutamate and/or GABA concentrations; however, to induce such long-lasting clinical effects, multiple sessions of cTBS or multiple doses of a pharmacologic agent would be required.

Of note, Ni and colleagues recently applied intermittent theta burst stimulation (iTBS) to bilateral posterior superior temporal sulci (pSTS) in adults with ASD and showed improvements in parent-reported ASD symptoms and compulsive behaviors (as measured by AQ and Y-BOCS scales) (76, 77), but no change in white matter macro/micro structure (78). However, when the same group applied cTBS to the dorsolateral prefrontal cortex (DLPFC), they did not find any significant improvement (as compared to sham stimulation) in executive functioning symptoms in a recently published study of children, adolescents, and adults with ASD (79). Taken together with the literature on aberrant functioning of the pSTC region and its relationship to ASD symptoms described above, Ni and colleagues findings suggest that the pSTC may be a better putative target than the DLPFC for future rTMS studies in ASD. Experimental therapeutics approaches and proof of mechanism studies such as the one described in the current paper will provide evidence for target engagement to guide the development of optimal parameters and protocols for future rTMS studies in ASD.

## 2 Methods

### 2.1 Study design

This study employs a within-subject crossover design whereby participants receive both active and sham cTBS on different days based on a randomized blinded code that is entered into the TMS machine before each session. The crossover design was chosen because of the documented heterogeneity of neuroimaging outcomes within the ASD population as well as the interindividual variability in response to cTBS and other rTMS protocols. Therefore, it was determined that a between-subject design would be less powerful in determining the acute effect of cTBS on these neuroimaging outcomes.

Participants, study staff members and all other study personnel involved in TMS administration, data collection, or data analysis are blinded to whether sham or active stimulation is delivered during each cTBS session.

### 2.2 Participants

This study was designed in two phases: A pilot phase (that included 20 healthy adult volunteers) and a main phase (that will include 86 adolescents with ASD).

**Figure 1:**
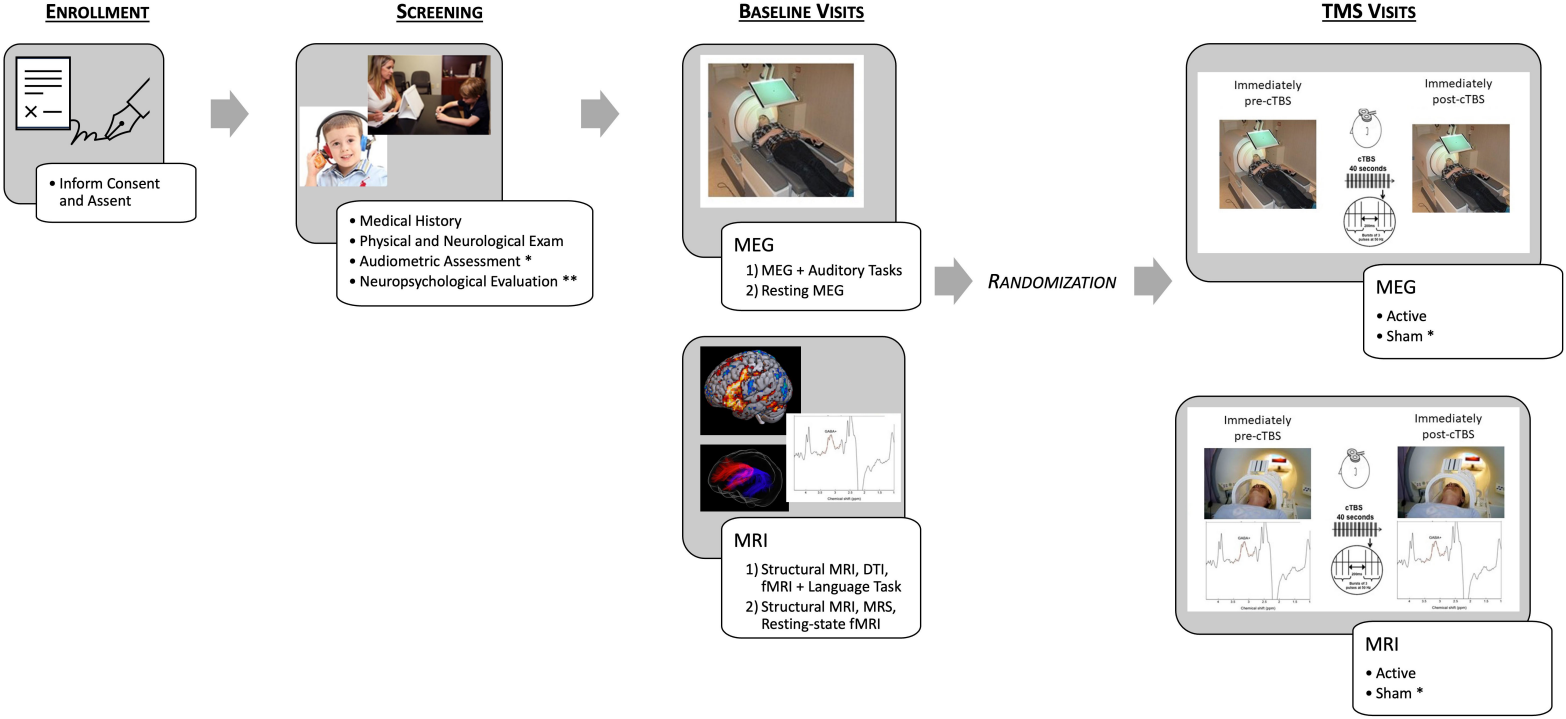
Schematic depicting the study protocol. After enrollment, participants complete screening, including an audiometric assessment and neuropsychological evaluation only for the individuals with ASD [Healthy Volunteer (HV) participants receive an abbreviated Neuropsychological Evaluation]. Baseline imaging was then conducted, and participants were randomized to either complete MEG/TMS or MRI/TMS sessions first and then within the session randomization active or sham (for ASD participants only) first. For example – MEG/active TMS, MEG/sham TMS, MRI/sham TMS, MRI/active TMS. * indicates procedures only completed by ASD participants. ** indicates procedures that differed between HV participants and ASD participants.

Inclusion criteria for the pilot phase were, (1) 18-25 years old; (2) No known neurological, psychiatric, genetic, or chronic uncontrolled general medical disorder. Exclusionary criteria for both phases include: (1) Known neurological, psychiatric, or general medical conditions in which MRI or rTMS might result in increased risk of side-effects or complications or might confound the results; (2) Individuals currently taking GABAergic medications or other medications that significantly lower seizure threshold; (3) Individuals with a previous history of rTMS. The pilot phase of the study included: baseline sessions during which MEG, DWI, resting-state and task-related fMRI, as well as MRS scans were acquired. Baseline sessions could be divided into multiple visits to reduce burden to the participant. Most individuals had three baseline visits, MRI baseline visit 1 (DWI and task-related fMRI), MRI baseline visit 2 (resting-state fMRI and MRS), and baseline MEG. Additionally, participants received behavioral/cognitive assessments and a thorough medical history and physical to screen for potential risk factors associated with side effects of rTMS. Participants eligible to continue then completed an active cTBS/MRI visit and an active cTBS/MEG visit (**Figure 1**). **Tables 1**, **2** summarize the descriptive characteristics and baseline imaging for those included in the pilot phase.

**Table 1:** Demographics for the Healthy Volunteer sample in the pilot phase.

N (Male/Female)	20 (5/15)
Age, years (Mean ± SD)	23 ± 0.94
FSIQ (Mean ± SD) SRS-2 Total T score (Mean ± SD) SRS-2 SCI T score (Mean ± SD) SRS-2 RRB T score (Mean ± SD)	121.3 ± 13.0 (n=18) 43.26 ± 4.30 (n=19) 44.58 ± 4.07 (n=19) 43.21 ± 4.48 (n=19)

Full Scale Intelligence Quotient (FSIQ) measured by Wechsler Abbreviated Scale of Intelligence, Second-Edition (WASI-II) with a population mean of 100 and standard deviation of 15. Two participants were withdrawn prior to completing this assessment. Social Responsiveness Scale, Second Edition (SRS-2) T scores are presented for the total scale, Social Communication Subscale (SCI), and Restrictive and Repetitive Behaviors Subscale (RRB). SRS-2 Total T scores of 59 and below are considered to be within typical limits.

**Table 2:** Baseline imaging measures for the Healthy Volunteer sample in the pilot phase.

Region	Measurement	Mean ± SD
Left pSTC	GABA/Cre Concentration (mmol/L)	0.54 ± 0.05 (n=10)
	Glutamate/Cre Concentration (mmol/L)	1.25 ± 0.13 (n=10)
Left Heschl’s Gyrus	Evoked Gamma Power	0.32 ± 0.38 (n=13)
	M100 Latency (ms)	98.4 ± 9.9 (n=10)
Right Heschl’s Gyrus	Evoked Gamma Power	0.48 ± 0.36 (n=13)
	M100 Latency (ms)	97.6 ± 7.7 (n=9)
Left Arcuate Fasciculus	FA	0.45 ± 0.01 (n=19)
	MD	0.76 ± 0.01 (n=19)
	RD	0.55 ± 0.02 (n=19)
	AD	1.14 ± 0.02 (n=19)
Right Arcuate Fasciculus	FA	0.45 ± 0.02 (n=19)
	MD	0.75 ± 0.04 (n=19)
	RD	0.55 ± 0.02 (n=19)
	AD	1.13 ± 0.02 (n=19)
Left Auditory Radiations	FA	0.39 ± 0.01 (n=19)
	MD	0.89 ± 0.04 (n=19)
	RD	0.71 ± 0.04 (n=19)
	AD	1.26 ± 0.04 (n=19)
Right Auditory Radiations	FA	0.39 ± 0.02 (n=19)
	MD	0.88 ± 0.02 (n=19)
	RD	0.70 ± 0.03 (n=19)
	AD	1.24 ± 0.03 (n=19)

GABA and Glutamate concentrations are measured over creatine. The MRS voxel was centered on the individualized cTBS target as defined by the peak bold activation to the ADDT task. Voxels were 2.5 cm cubed, thus the value represents relative concentrations of metabolites within the posterior superior temporal cortex.

Mean and standard deviation values for Evoked Gamma Power and FA are dimensionless.

Mean and standard deviation values for MD, RD, and AD are measured in 10^-3^ mm^2^/s.

Sample sizes varied based on data quality and visit completion.

FA, Fractional Anisotropy; MD, Mean Diffusivity; RD, Radial Diffusivity; AD, Axial Diffusivity.

For the main phase, ASD individuals must meet the following inclusion criteria: (1) Age 14-17, (2) Community diagnosis of ASD based on DSM-IV or DSM-5 criteria, (3) FSIQ > 70, (4) Right-handed, and (5) Normal hearing. In addition to the exclusion criteria listed above for the pilot phase of the study, in the main phase individuals with known genetic disorders associated with the ASD diagnosis or that in the opinion of the investigator may increase the risk to the participant or compromise the integrity of the data will also be excluded. In the main phase of the study, we will acquire the same baseline assessments as in the pilot phase as well as additional behavioral/neuropsychological assessments related to their ASD diagnosis and their receptive and expressive language skills. Participants will also complete an audiometric evaluation to confirm that they have normal hearing. To reduce burden to the participant these baseline visits can be further divided. Additionally, each ASD participant will receive both active and sham cTBS on two separate sessions, combined with MRI and combined with MEG for a total of four imaging/cTBS visits instead of the two in the pilot phase.

### 2.3 Power analyses and sample size determination

The first aim of the study is to establish the relationship between local GABA and glutamate neurotransmitter concentrations, structural and functional network connectivity, and physiological measures of auditory/language network functioning. The estimated effect size for this aim is approximately 0.2223. This is estimated based on the literature showing a significant correlation between GABA+/Cre concentration and MEG evoked gamma power that range from 0.37-0.53 (49, 52, 56, 80). To our knowledge there is not any existing data on the correlation between the other predictor variables (DWI or functional MR connectivity) and MEG power and latency of evoked fields. However, given that both of these neuroimaging predictor variables have been shown to be aberrant in adolescents with ASD and reflect network-level structural and functional dysfunction, we predict a correlation between these neuroimaging measures and the proposed MEG measure of at least 0.15. Thus, in order to maintain 80% power with a type-I error rate of 5% and a type-II error rate of 20% we will enroll 86 ASD participants with a planned evaluable N=55 accounting for attrition.

The second aim of the study is to evaluate target engagement and the acute effects of active cTBS compared to sham cTBS on auditory/language network functioning. With planned evaluable N=55 and the minimal detectable effect (given Lehr’s equation (81)), the minimum detectible effect size is 0.38 standard deviation units, between a small (.2) and medium (.5) effect size in Cohen’s effect size taxonomy (82). We believe this is a reasonable effect size to expect given the literature. A recent study found an effect size of 0.7 on MRS GABA+/Cre concentration and 0.39 on MEG indices of sensory (visual) processing immediately following a single session of cTBS [95]. Therefore, we believe this aim is adequately powered.

### 2.4 Procedures

#### 2.4.1 Medical, neuropsychological, and neurodevelopmental assessments

After completing informed consent, all participants (across both phases of the study) receive a thorough medical history and brief neurological and physical exam by a licensed physician or nurse practitioner, where concomitant medications are also reviewed. Participants then complete several standardized neuropsychological and neurodevelopmental assessments and rating scales. The Social Responsiveness Scale (SRS-2) and Wechsler Abbreviated Scale of Intelligence (WASI-II) were completed in the pilot and main phases, and the remaining measures listed below will be administered only to participants in the main phase of the study (ASD).

##### 2.4.1.1 The social responsiveness scale, second edition

The SRS-2 is a 65-item scale completed by the Healthy Volunteer or the parent/caregiver on behalf of their child and is designed to screen children and adults for autistic traits (83). There is a self-report and a parent/caregiver-report form, in which the individual completing the form rates each item based on a 4-point Likert scale from ‘not true’ to ‘almost always true’ regarding their (for the Healthy Volunteers)/their child’s (for the parent/caregiver of the ASD participant) behavior in the previous six months. Items within each of the two subscales and a composite score are calculated into a standard raw score and T-score.

##### 2.4.1.2 The child behavior check list

The CBCL is a 118-item scale completed by the parent/caregiver on behalf of their child and is designed as a standardized assessment of children’s symptomatology across several domains including anxiety, attention problems/hyperactivity, conduct problems, depression, oppositional defiant, social problems/immaturity, and somatization (84). The individual completing the form rates each item based on a 3-point Likert scale (“Not True”, Somewhat or Sometimes True” and “Very True or Often True”) regarding their child’s behavior in the previous six months. A total score and subscale scores (corresponding to specific symptom/DSM diagnostic domains) are calculated.

##### 2.4.1.3 Wechsler abbreviated scale of intelligence, second edition

The WASI-II is a brief, reliable measure of general cognitive ability as well as a verbal comprehension and perceptual reasoning index score (85). It is appropriate for use in children and adults ages 6-90. Healthy Volunteers and Adolescents with ASD will be included if their FSIQ>70, thus excluding participants with intellectual disability.

##### 2.4.1.4 Vineland adaptive behavior scales-3^nd^ edition

The VABS-3 is a semi-structured parent/caregiver interview designed to evaluate adaptive functioning of their child in four domains: Communication, Daily Living Skills, Socialization, and Motor Skills (86). Standard scores are provided for each domain (with the exception of Motor Skills for children over age 9) and for an overall Adaptive Behavior Composite Score.

##### 2.4.1.5 The autism diagnostic interview-revised

The ADI-R is a structured interview conducted with the parents/caregivers of individuals with ASD (87). It covers their child’s full developmental history and generally takes one to two hours. Parents/Caregivers are asked 93 questions about either their child’s current behavior or behavior at a certain point in time. The interview is divided into four main sections assessing the quality of social interaction, language and communication, restricted and repetitive behaviors, and maladaptive behaviors such as self-injury, aggression, and over activity. The interview determines a rating score for each question, and a total score is calculated for each of the content areas. For the purposes of this study, ADI scores are used as a baseline measure for exploratory analyses. Eligible participants for the main phase of the study have received an ASD community diagnosis per DSM-IV or DSM-V criteria.

##### 2.4.1.6 Expressive vocabulary test, third edition

The EVT-3 is an individually administered, norm-referenced instrument that assesses expressive vocabulary and word retrieval for children and adults (88). The test contains training items and 190 test items arranged in increasing difficulty. For each item, the examiner presents a picture and reads a stimulus question, and the examinee responds with one word that provides an acceptable label, answers a specific question, or provides a synonym for a word that fits the picture.

##### 2.4.1.7 Peabody picture vocabulary test, fifth edition

The PPVT-5 is a norm-referenced instrument for measuring receptive (hearing) language processing (89). It is appropriate for use in children and adults as young as 2.5 years old. The test contains training items and 228 test items, each consisting of four full-color pictures as response options on a page. For each item, the examiner says a word, and the examinee responds by selecting the picture that best illustrates that word’s meaning.

##### 2.4.1.8 Clinical evaluation of language fundamentals

The CELF-5 is a standardized assessment of language used to diagnose language disorders in individuals 5 to 21 years of age (90).

#### 2.4.2 Imaging acquisition procedures

##### 2.4.2.1 MEG

MEG Data is acquired using a sampling rate of 1200Hz on a 275-channel MEG (CTF MEG NEURO INNOVATIONS, INC.) system. During acquisition, participants lay in the supine position on a comfortable bed with their head in the sensor helmet. Three fiducial marker coils are placed relative to anatomical landmarks: 1.5 cm superiorly to the nasion and 1.5 cm anteriorly to the left and right periauricular points along the line between the tragus and lateral canthus. These coils are activated at the beginning and end of the acquisition to localize the head within the MEG device and coarsely assess head movement during the scan. Directly prior to data acquisition, the localizer coils are registered to the anatomical MRI using Brainsight Neuronavigation software (Rogue Research, Canada). Both resting-state and task data are acquired. Prior to data acquisition hearing thresholds are determined by presenting 1000 Hz tones (300 ms duration, 10 ms rise time) to each ear sequentially and monotonically decreasing loudness until sensation level (SL) has been determined for each ear. Sounds for the MEG tasks are presented at approximately 45 decibels above SL. MEG recording sessions last ~45 minutes, and consist of the following paradigms:

1. An M100/M50 task, consisting of binaurally presented 500Hz tones, with a variable inter-trial interval (ITI) using the task reported in Roberts et al. (91). Note, there is a developmental trajectory in response to these stimuli with adults showing a prominent evoked field peak around 100 milliseconds, with an absent and/or less reliable peak at 50 milliseconds (92, 93) and the opposite (a more pronounced 50 millisecond peak) in children and especially those with ASD (94).
2. Auditory 40 Hz steady-state task (ASSR), presenting 200 trials of 40 Hz amplitude modulated (AM) 500Hz sinusoidal tones (500 ms long, 45 dB SL) with a jittered 1.5 sec inter-stimulus interval as in Edgar et al. (63),.
3. Resting MEG: 5 minutes eyes open in a completely dark room (with short periods of interspersed dim light) and 5 minutes eyes closed in a lighted room (with short periods of interspersed eyes open) as in Edgar et al. (95). Resting MEG was only collected in the eyes closed lighted room condition for the pilot phase.
4. Oddball auditory task with interleaved/a/and/u/vowel stimuli as in Roberts et al. (96).

##### 2.4.2.2 Structural MRI

All MRI and MRS data are acquired on a General Electric (GE) MR-750 3T scanner using a 32-channel head coil. For targeting of the TMS and registration of the fMRI, DWI, and MRS scans, the following structural MRI sequences are acquired (1): A 6-minute high-resolution T1 weighted Magnetization Prepared Rapid Gradient Recalled Echo (MP-RAGE) with repetition time (TR) = 7.7 ms, echo time (TE) = Min Full, flip angle = 7 degrees, a spatial resolution of 1.0 × 1.0 × 1.0 mm^3^, and 176 slices; (2) A 1.5-minute T1 weighted Spoiled Gradient Recalled Echo (SPGR) with TR = 200 ms, TE = In phase, flip angle = 80 degrees, a spatial resolution of 0.8 × 0.9 × 3.0 mm^3^, and 20 slices; (3) A 2.5-minute fat suppressed T2 weighted with TR = 6553 ms, TE = 100 ms, flip angle = 125 degrees, a spatial resolution of 0.9 × 1.2 × 1.7 mm^3^, and 100 slices; (4) For electrical-field (EF) modeling, a 5.5-minute (nonfat-suppressed) T2 weighted with TR = 2500 ms, TE = Maximum, a spatial resolution of 1.0 × 1.0 × 1.0 mm^3^ and 176 slices. Electric field (e-field) modeling has been added to the main phase to optimize placement of the TMS coil.

##### 2.4.2.3 MRS

To measure the relative concentrations of metabolites (N-acetylaspartate, creatine plus phosphocreatine, choline-containing compounds, GABA, and glutamate/glutamine) and metabolite ratios, a magnetic resonance spectroscopy J-editing sequence (45) is used with TR = 1500 ms, TE = 68 ms, and voxel size 2.5 cm^3^ for a volume of 15.6ml (close to the default of 18 ml) (45) The GABA levels are measured with an adapted version of GE’s standard PRESS sequence PROBE. The addition is a set of editing pulses that act upon resonances of 1.9 ppm and higher (45). Creatine at 3 ppm is not affected by the editing pulse but the GABA triplet at 3.1 ppm is, after a subtraction the GABA signal remains. The number of data points is 4096, sampled at 5 kHz, and the number of averages is 784 with in scanner averages NEX=2. After the scan 16 non-water-suppressed reference scans are made, resulting in a total scan time of 20 minutes.

An anatomical region of interest (ROI) is defined based on a rotated T-1 weighted anatomical scan obtained during the same scanning session, according to standard anatomical atlases. Prior to the scan, a vitamin E capsule is placed on the scalp region corresponding to the individually defined cTBS target within the left pSTC using Brainsight Neuronavigation software (Rogue Research, Canada). The voxel is placed in a rotated anatomical scan with the center in line with a vitamin E capsule during each scanning session. The voxel size was chosen to be 2.5 cm^3^ to match the approximate spread of the electric field induced by the TMS stimulation (97). To prevent lipid contamination of the skin and to cover the brain tissue underneath the skull as close as possible, the voxel is placed in a rotated anatomical scan over the anterior-posterior and inferior-superior axes, such that the face of the cubic voxel is parallel to the skull at the placement of the capsule. High bandwidth saturation pulses are used to saturate lipid signals originating from tissue close to the six faces of the cube.

##### 2.4.2.4 DWI

To quantify white-matter microstructural indices of network connectivity, diffusion weighted imaging (DWI) data is acquired employing a 2D spin-echo echo-planar imaging (SE-EPI) sequence. Two scans (13 minutes each) are acquired in anterior–posterior (AP) and posterior–anterior (PA) phase-encoding directions. We use a high-angular resolution diffusion imaging (HARDI) acquisition scheme with b-values of 0 s/mm^2^ (10 gradient directions), 300 s/mm^2^ (10 gradient directions), and 1100 s/mm^2^ (60 gradient directions). Other sequence parameters include: TR = 9667 ms, TE = Minimum, slice thickness = 2 mm, 80 axial slices, a spatial resolution of 2 mm^3^.

##### 2.4.2.5 fMRI

For identifying an individualized TMS target and quantifying task-based BOLD activation and task-based functional network connectivity, a 5-minute language localizer functional scan is acquired with TR = 2000 ms, TE = 30 ms, flip angle = 65 degrees, a spatial resolution of 3.0 × 3.0 × 3.0 mm^3^ and 40 slices. During the scan, participants are asked to perform an Auditory Description Decision Task (ADDT). The task consists of two block types: (1) a series of beeps interspersed within “non-sensical” sounds (English statements pronounced backward) and (2) true (correct target) and false (foil) statements (e.g., a true statement would be “Something that hangs in a museum is a painting.” And a false statement would be “Something that parts your hair is a refrigerator.”). There are a total of ten 30-second alternating blocks, beginning with block type 1. Participants are instructed to push a button when they hear a beep (block type 1 – beeps and sounds) and when the description is true (block type 2 – true/false statements). Twenty of 30 items are correct targets; 10 of 30 are foils. This task has been shown to reliably activate both superior temporal as well as inferior frontal language regions (98–100) and is recommended, based on its reliable, robust activation of language areas, for presurgical language mapping (101).

In addition to the task-related functional scan, a resting-state fMRI scan is acquired for resting-state functional connectivity analyses. During the pilot phase, the resting-state scan was acquired with a 12-minute single-shot, partially parallel, gradient-recalled echo planar sequence with sensitivity encoding, TR = 2000 ms, TE = 30 ms, flip angle = 65 degrees, a spatial resolution of 3.0 × 3.0 × 3.0 mm^3^, and 40 slices. Resting-state functional connectivity for the main phase is measured using a 12-minute hyperband, gradient-recalled multi-echo (ME = 3) sequence with TR = 2000 ms, TE = Min Full, flip angle = 65 degrees, a spatial resolution of 3.0 × 3.0 × 3.0 mm^3^, and 48 slices. A fixation cross is provided during this scan. Participants are instructed to keep their eyes open, remain as still as possible, and “think of nothing in particular”.

#### 2.4.3 Continuous theta burst stimulation

As shown in **Figure 1**, each participant in the pilot phase completed two active cTBS sessions (one with MRI and one with MEG acquired immediately before and after stimulation). Participants in the main phase of the study will complete four cTBS sessions (two active and two sham). cTBS sessions are scheduled approximately once/week to avoid carry-over effects from one visit to the next. The cTBS protocol consists of bursts of three pulses of 50 Hz stimulation repeated at 200 ms intervals (5 times per second) for 40 seconds (for a total of 600 pulses). Stimulation is applied at an intensity of 80% of active motor threshold (AMT) defined as the minimum stimulator intensity required to elicit a motor evoked potential from the first dorsal interosseous muscle of at least 200 microvolt peak to peak amplitude on five out of ten trials while the participant maintains voluntary contraction of the target muscle. A neuronavigation system (Brainsight, Rogue Research, Canada) is used to track the position of the TMS coil over the stimulated target. The target is defined by combining e-field modeling (described below) with the participant’s peak BOLD activation within the left pSTC during the baseline ADDT task, overlaid on the structural anatomical scan. A functional localizer (rather than a single structurally-defined ROI) was used in this study as previous literature suggests significant interindividual variability in both typically developing individuals (102) as well as those with neurological disorders (103, 104). The feasibility of the application of TBS in the proposed study populations has also been well supported in the literature and by our own experience (65, 105–107).

In the current study, cTBS is administered using a MagPro X100 magnetic stimulator (P/N: 9016E711, Magventure A/S) and a double-sided Cool-B65 Active/Placebo (A/P) coil (P/N: 9016E0501, MagVenture A/S). Inside the A/P coil, there is a figure-of-eight winding on the active side and a metal shield on the sham side, separated by approximately 8 cm. For active stimulation, the active side of the coil is placed on the scalp; for sham stimulation, the coil is flipped, and the spacer and shield attenuate the field reaching the brain. Visually, both sides of the coil are identical, therefore blinding the TMS technician to active or sham mode. During a cTBS session, a unique patient code, supplied by MagVenture, is inputted into the MagPro X100 system, which then prompts the TMS technician to either flip or maintain coil orientation. A randomized schedule of visits was created using block randomization and the unique patient codes designating active or sham mode, and is kept in an electronic password protected document by an unblinded study staff member. The order of sessions is scheduled to ensure that the participant receives active and sham stimulation for one modality (MRI or MEG) prior to moving on to the other modality. Thus, there are only four potential orders (Active, Sham, Sham, Active; Active, Sham, Active, Sham; Sham, Active, Sham, Active; and Sham, Active, Active, Sham). The block randomization ensured that there will be the same number of participants receiving the cTBS in each of these orders.

Participants are asked to complete an Assessment of Blinding following each cTBS session to assess the integrity of the blind. During the assessment, participants are asked to guess whether they received active or sham TMS and to report their confidence in their guess on a scale from 0 (not confident at all) to 5 (extremely confident). Participants are also asked to rate the loudness and painfulness of the stimulation and to describe any facial muscle twitching or additional side effects experienced during cTBS.

#### 2.4.4 Image processing procedures

##### 2.4.4.1 MEG

Data is converted to MEG-BIDS (108) format using MNE-BIDS toolbox (109) and is analyzed with MNE-Python toolbox (110) for electrophysiological research, Freesurfer (111) for anatomical surface preparation, and custom scripts written in Python and Bash. The raw MEG data sets (baseline, and post stimulation for active TBS and sham TBS) for each participant undergo quality control procedures to remove trials corrupted by artifacts. Broadband evoked responses are extracted, as well as induced changes in oscillatory power in the time frequency response. Data are analyzed in both sensor space and anatomical source space. Forward modeling of the down-sampled cortical manifold is assessed using a realistic single layer boundary element model and source localization is performed using beamformer or minimum norm techniques, depending on the task. For evoked responses, source localized time series amplitude and latency (defined as the time interval between stimulus and maximal amplitude) are determined. For induced power analyses, the peak induced gamma frequency will also be determined. In the context of the M100/M50 task, two components are identified – the M50 and the M100, both occurring in Heschl’s gyrus, in left and right superior temporal cortices. In the speech processing oddball paradigm, the mismatch negativity is identified as the difference between the evoked responses to the frequent and rare tones. For the auditory steady state experiment, inter-trial coherence is calculated as described in (112).

##### 2.4.4.2 MRS

Custom software is used to measure the relative concentrations of metabolites (N-acetylaspartate, creatine plus phosphocreatine, choline-containing compounds, GABA, and glutamate/glutamine) and metabolite ratios. The data is pre-processed with an eddy current correction and the data is tested for patient movement by tracking the residual water signal, edit - non edit pairs with residual water deviations larger than 10% are rejected (45). Subsequently, the averages of the data are frequency and phase corrected before summation (113). In a last step, the summed data is fitted with simulated reference signals for the non-edited, the edited, and the subtracted data (113). Standard statistical packages such as Statistica are used to determine significant differences in the metabolites taken immediately prior to and immediately following cTBS administration. In-house software was written to derive ratios of GABA and glutamate to creatine.

##### 2.4.4.3 DWI

DWI data undergo processing using multiple software tools, including MRtrix3 (114), FSL (115), Freesurfer (116), ANTS (117), and TractSeg (118). The anterior–posterior (AP) and posterior–anterior (PA) phase-encoding scans are concatenated. Marchenko-Pastur principal component analysis (PCA) are used to denoise the DWI data via the MRtrix3 *dwidenoise* function, followed by removing Gibbs ringing artifacts using the MRtrix3 *mrdegibbs* function. Eddy current correction are performed using the MRtrix3 *dwifslpreproc* function, utilizing the FSL linear second-level model (*–slm=linear*) and the default outlier replacement (*–repol*) parameters. Additionally, bias field correction is applied using both MRtrix3 and ANTs software *dwibiascorrect ants*. To increase anatomical contrast and improve tractography results, the DWI images are up-sampled to an isotropic voxel size of 1.25 mm using *mrgrid*. Whole-brain masks are generated from the up-sampled images using FSL Brain Extraction Tool 2, *bet2*, with a 0.3 fractional intensity threshold. The basis function for multi-tissue types (white matter (WM), gray matter (GM), and cerebrospinal fluid (CSF)) are derived using Dhollander’s algorithm, *dwi2response dhollander*, to estimate the fiber orientation densities (FODs) within each voxel. To perform multi-shell and multi-tissue analysis, the basic functions for each tissue type are applied to the DWI data using the MRtrix3 *dwi2fod msmt_csd* function. For group-level analysis, the FODs are normalized. The peaks of the WM FODs are extracted from each voxel using *sh2peaks*.

To investigate specific white matter tracts, the left and right arcuate fasciculus and auditory radiation fiber bundles are extracted using the WM peaks with an automatic fiber tracking package, TractSeg, in conjunction with FSL XTRACT atlas (119). TractSeg uses deep machine learning algorithms to extract these fiber bundles. Finally, a tensor model is fitted using *dwi2tensor* and the fractional anisotropy (FA), axial diffusivity (AD), radial diffusivity (RD), and mean diffusivity (MD) maps are created using *tensor2metric* and average values are extracted for specific tracts.

##### 2.4.4.4 Task-based fMRI

**Figure 2:**
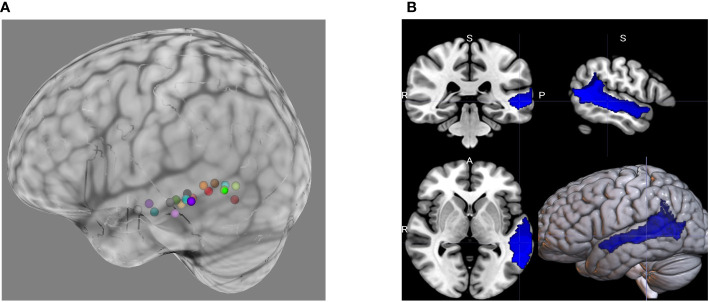
**(A)** Individualized cTBS targets during the pilot phase of the study were based on peak BOLD activation during the ADDT, a language task. The choice of cTBS target will be constrained during the ASD main phase of the study. This will be done using **(B)** a mask generated from merging seven individual ROIs from the Sensaas atlas (e.g., SMG7, T1_4, STS2, STS3, STS4, T2_3, T2_4).

Afni (version 22.3.05) (120) is used to process the task-based fMRI data, afni_proc.py is used to setup a full pipeline for the fMRI analysis of each participant, including the automatic generation of a quality control HTML for evaluating the data and processing steps (121). The first 14 TRs (28 seconds) are discarded from the analysis to allow stabilization of the magnetic field and noise cancellation. All images are corrected for slice acquisition timing, motion corrected to the minimum outlier volume, and spatially smoothed with a 4mm full-width-half-maximum smoothing kernel. EPI-anatomical alignment is performed using the lpc+ZZ cost function (122) with local EPI unifizing for additional stability, and these datasets are checked for left-right consistency (123). To reduce effects of participant motion, volumes with large motion (Enorm > 0.3 mm between successive time points) are censored. A block regression basis is used, in which the onset of the true/false statement blocks are defined. The contrast between those blocks (block type 2) and implicit baseline (block type 1 – beeps and sounds) are computed. Several steps are then performed for quality control evaluation such as checking the alignment between the anatomical, EPI, and template image; checking that the stimuli are properly assigned between each stimulus class; and checking how many data points are censored because of motion. The final language (block type 2) > implicit baseline (block type 1) statistical map and the participant anatomical scan are then loaded into the Brainsight Neuronavigation system (Rogue Research, Canada). The 3dClusterize command is then used on the t-statistical map to extract the coordinates of the peak activation in the Wernicke area for each participant and is chosen as the cTBS target. For the ASD main phase of the study, a mask will be used to constrain the choice of the stimulation target. The mask was generated by selecting seven ROIs from the Sensaas atlas (https://github.com/loiclabache/SENSAAS_brainAtlasL: SMG7, T1_4, STS2, STS3, STS4, T2_3, T2_4), and merging them into a single ROI (see **Figure 2B**). E-field modeling is an additional step for the main phase and will be added to further refine the cTBS target and optimize delivered dosage. The induced e-field with the figure of eight coil used in the current study is estimated to be approximately 2.5 cm^3^ (97).

To perform e-field modeling, first, the mri2 mesh pipeline within the Simulation of Noninvasive Brain Stimulation (SimNIBS) version 3.2.1 software (124) is used to construct computational head models to determine the induced E-field at the individualized target based on the peak BOLD activation from the task. Then, the targeting and analysis pipeline (TAP) software (125) add-on is used to determine the optimal TMS coil placement on the scalp. A voxel containing the individualized target in an otherwise empty MRI image file is inputted into the TAP software. The TAP software uses the computational head model generated by the SimNIBS and determines the closest point in the sulci wall and its normal vector. This normal vector, which is perpendicular to sulci wall, is used in SimNIBS to find the TMS coil placement which maximizes the E-field going into the sulci wall known to cause the best stimulation effect (126). The search for this optimal TMS coil placement is performed on the scalp surface grid (accounting for 2mm hair thickness) with 1 mm edge length (1-degree TMS orientation increments) in a circular area (with radius of 25 mm) around the scalp-projected point of the coordinate. This optimal coil setup is saved to a text file and is then used with the Brainsight Neuronavigation system (Rogue Research, Canada) during the session.

We also perform a psychophysiological interaction analysis with the CONN toolbox to study the functional connectivity changes, using the same seed regions. For each pair of seed and target areas, a generalized psychophysiological interaction model (gPPI (127, 128)) is defined with seed BOLD signals as physiological factors, boxcar signals characterizing each individual task condition convolved with an SPM canonical hemodynamic response function as psychological factors, and the product of the two as psychophysiological interaction terms. Functional connectivity changes across conditions are characterized by the Fisher-transformed semi partial correlation coefficient of the psychophysiological interaction terms in each model. Finally, group-level analyses are performed using a General Linear Model (GLM (129)). For each individual voxel a separate GLM is estimated, with first-level connectivity measures at this voxel as dependent variables (one independent sample per subject and one measurement per experimental condition), and groups or other subject-level identifiers as independent variables. Voxel-level hypotheses are evaluated using multivariate parametric statistics with random-effects across subjects and sample covariance estimation across multiple measurements. Inferences are performed at the level of individual clusters (groups of contiguous voxels). Cluster-level inferences are based on parametric statistics from Gaussian Random Field theory (130, 131). Results are thresholded using a combination of a cluster-forming p < 0.001 voxel-level threshold, and a familywise corrected p-FDR < 0.05 cluster-size threshold (132).

##### 2.4.4.5 Resting-state fMRI

Analysis of functional connectivity between predefined ROIs within the dorsal and ventral language networks (i.e. pSTC, posterior inferior temporal cortex, temporal-parietal junction, and inferior frontal gyrus) is performed using CONN toolbox (133) (RRID : SCR_009550) release 22.a (134) and SPM (135) (RRID : SCR_007037) release 12.7771.

Functional and anatomical data are preprocessed using a flexible preprocessing pipeline (136), including realignment, outlier detection, direct segmentation, MNI-space normalization, smoothing, and denoising. For resting state connectivity, seed-based connectivity maps (SBC) and ROI-to-ROI connectivity matrices (RRC) are estimated with 164 HPC-ICA networks (134) and Harvard-Oxford atlas ROIs (137). Functional connectivity strength is represented by Fisher-transformed bivariate correlation coefficients from a weighted general linear model (weighted-GLM (138)), defined separately for each pair of seed and target areas, modeling the association between their BOLD signal time series.

### 2.5 Statistical analysis

Multiple linear regressions will be conducted to evaluate whether baseline MRS measures of local GABA and glutamate concentration, structural topography/connectivity, and/or functional activity/connectivity explain a significant proportion of the variability in MEG measures of auditory/language processing. These analyses will quantify the relationship between these measures and to what extent they may contribute to a fusion biomarker model for language processing deficits in ASD (e.g., (139)). To evaluate the effect of a single session of cTBS on MRS, fMRI, and MEG outcome measures, Factorial ANCOVAs will be conducted with time-point (pre-post) and condition (active, sham) as within-subjects variables. Other anticipated covariates in these analyses include age, gender, baseline symptom severity, and medication use. In addition to the targeted region (left pSTC), we will repeat the planned fMRI BOLD and connectivity analyses using right pSTC, left and right IFG, and a non-connected (control) cortical visual network with a temporal and frontal node to explore the spread of the effect of the cTBS from the targeted region to other structurally and functionally connected regions compared to a control network. Pearson correlation coefficients will be used for the exploratory analyses, to characterize the relationship between the neuroimaging and electrophysiological indices and baseline behavioral symptom presentation including assessments of general functioning, autism symptom severity, and auditory and language processing. As there may be intrasubject variability in these measures (independent of the cTBS intervention), we will evaluate the reliability of the baseline/pre-stimulation MEG, MRS, and fMRI outcomes by calculating the interclass correlation coefficient (Model: 2-way mixed effects, Type: mean of k measurements and Definition: absolute agreement) as a measure of test-retest reliability.

## 3 Anticipated results

The pilot phase of the study, completed in August of 2022, supported the feasibility and validity of our procedures and primary outcomes. Task-related fMRI demonstrated robust significant BOLD activation within the pSTC ROI as well as frontal cortical language-relevant regions during the ADDT task. Consistent with previous literature (102–104), the exact location of peak activation within the broader pSTC region varied between Healthy Volunteer participants, justifying individualized targeting for the main phase of the study. **Figure 2A** shows the individualized targets based on peak BOLD activation during the ADDT task for the participants in the pilot phase. Additionally, the MEG tasks evoked robust and reliable evoked fields and glutamate and GABA levels were able to be quantified using the MRS sequences described above.

**Figure 3:**
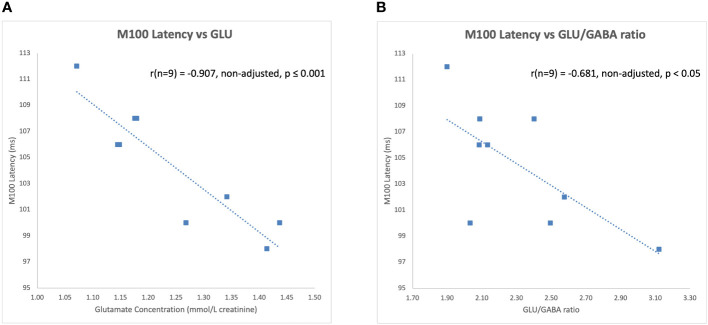
Left hemisphere M100 latencies negatively correlated with **(A)** glutamate (GLU) concentrations and **(B)** glutamate/GABA ratios at baseline during the pilot phase.

**Figure 4:**
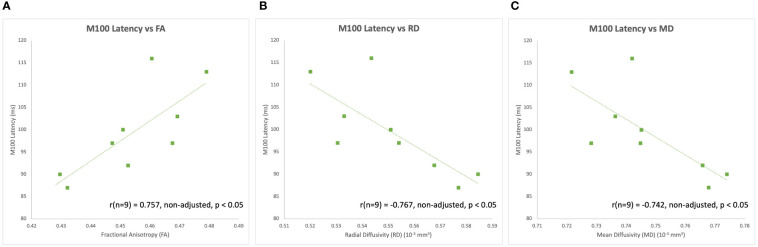
Left hemisphere M100 latencies positively correlated with **(A)** FA of the left Arcuate Fasciculus (i.e., left pSTC to left IFG) at baseline during the pilot phase. Left hemisphere M100 latencies also negatively correlated with **(B)** RD and **(C)** MD of the left Arcuate Fasciculus at baseline during the pilot phase.

**Figure 5:**
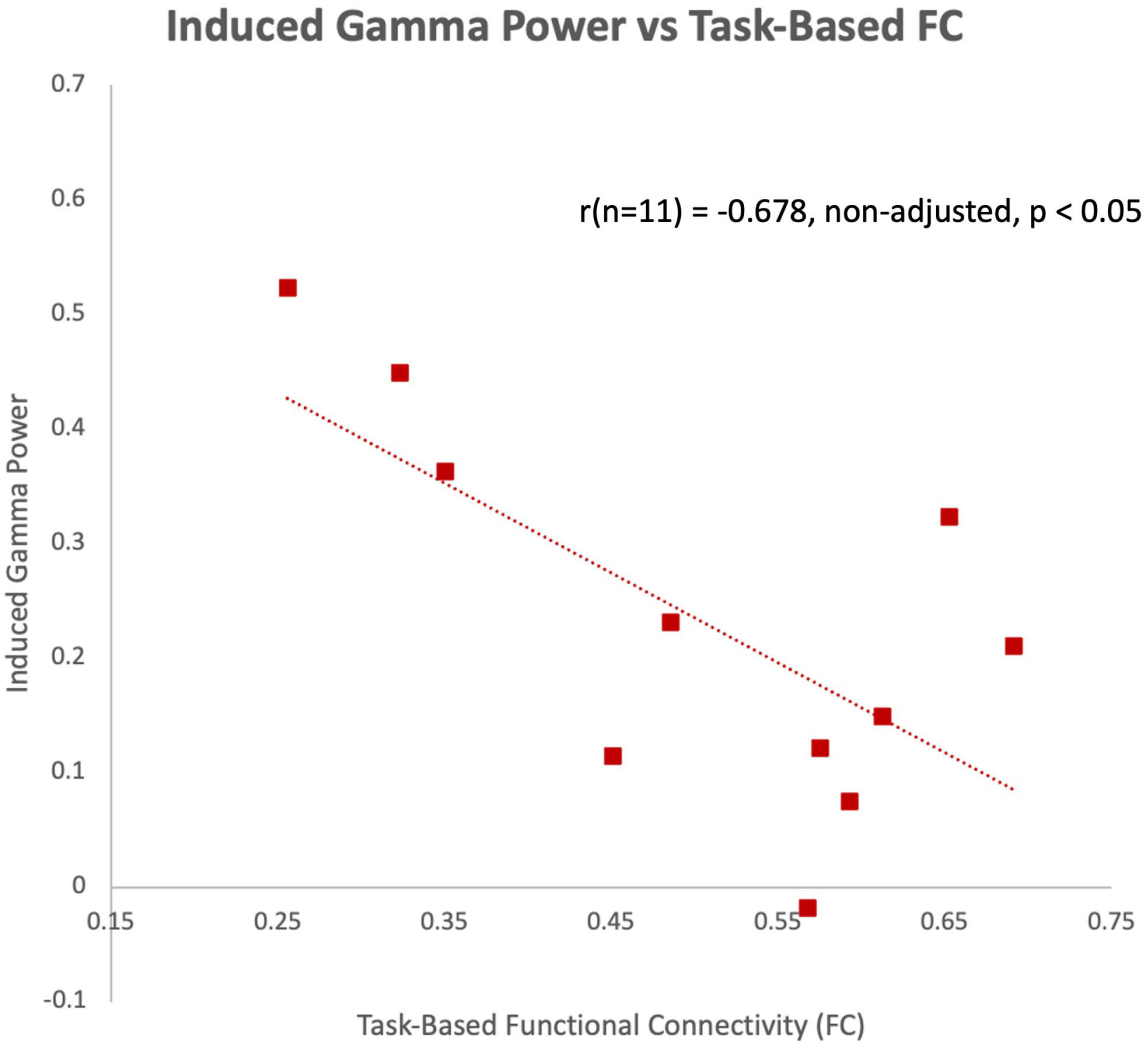
Induced gamma power of the ASSR in the left hemisphere negatively correlated with task-based functional connectivity between left pSTC and left IFG individualized seeds at baseline during the pilot phase.

**Figure 6:**
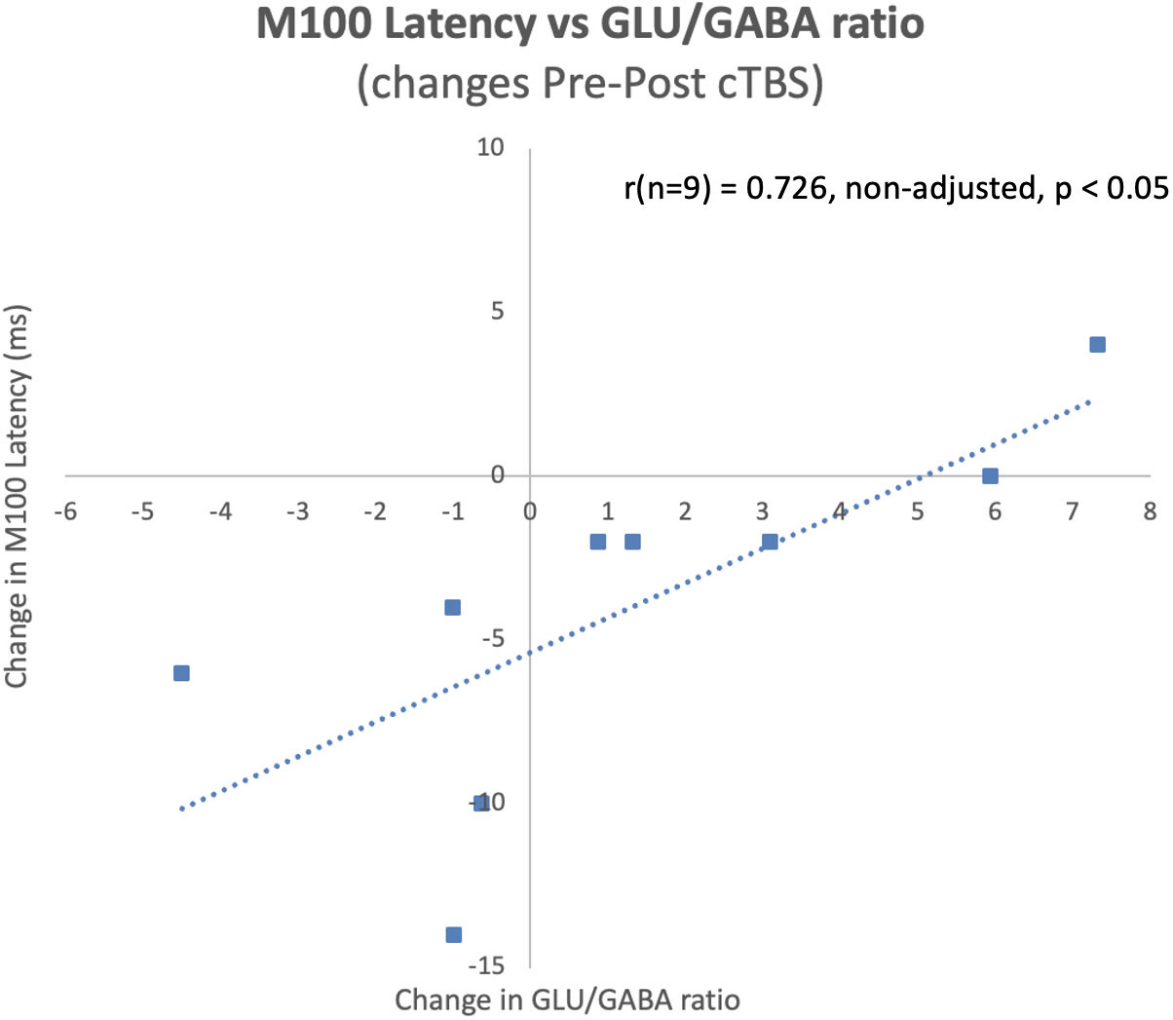
Pre-post cTBS changes in M100 latencies significantly correlated with pre-post cTBS changes in glutamate/GABA ratios at baseline during the pilot phase. Notably, these changes occurred in the predicted direction, where a decrease in the ratio of glutamate/GABA was observed for individuals who experienced a decrease in M100 latency.

The latency of the left hemisphere M100 MEG evoked field negatively correlated with baseline MRS glutamate (r (n=9) = -0.907, p<0.001) (**Figure 3A**) and glutamate/GABA ratios (r(n=9) = -0.681, p,0.05) (**Figure 3B**), positively correlated with Fractional Anisotropy (DWI metric) (r (n=9) = 0.757, p<0.05) (**Figure 4A**), and negatively correlated with Radial and Mean Diffusivity (DWI metrics) of the Arcuate Fasciculus (left pSTC to left IFG) (Radial Diffusivity: r(n=9) = -0.767, p<0.05; Mean Diffusivity (r(n=9) = -0.742, p<0.05) (**Figures 4B, C**). The induced power in the gamma frequency band (ASSR Task) at baseline correlated with baseline task-based functional connectivity between left pSTC and left IFG individualized seeds (r(n=11) = -0.678, p<0.05 (**Figure 5**). The single session of cTBS also led to changes in MEG and MRS measures in the predicted direction (e.g., Those individuals who experienced a decrease in glutamate/GABA ratio, also experienced a decrease in M100 latency) (**Figure 6**). Note that since the pilot phase participants were adults, as expected they did not display a reliable M50 evoked field. However, the main phase participants, as they will be adolescents, are expected to show an M50 and M100 evoked field to the stimulus. Thus, both the M50 and M100 evoked field will be evaluated in the main phase.

The pilot phase was not powered to achieve significance in these measures, however, the significant findings presented above provide preliminary data supporting our hypothesis of a relationship between measures of local neurochemical concentrations, structural/functional connectivity of the network, and MEG measures of auditory processing and the impact of a single session of cTBS on these measures. Brain functioning in typical development is presumably optimized, making further improvement or speed of basic auditory and language network functioning difficult to achieve (ceiling effect). Thus, there may have been a limit to how much gamma power could be evoked by the MEG task or how functionally connected a network could be during the fMRI task. Additionally, the directionality of these findings (positive versus negative relationships) may be different in the ASD group compared to this typically developing pilot sample.

In the ASD group, we hypothesize that the auditory and language networks are not optimized, thus, there will be more opportunity for modulation in these individuals (i.e., a ceiling effect is not expected in the ASD group). Based on these preliminary findings as well as the extant literature, we anticipate that baseline MRS glutamate and GABA concentrations, DWI measures of tissue microstructure, and task-related functional connectivity will be significant predictors of MEG indices of auditory/language processing in adolescents with ASD. Furthermore, we hypothesize that a single session of active cTBS compared to sham cTBS over the individually targeted region of the pSTC will lead to significant decreases in the glutamate/GABA ratio as measured by MRS, significant modulation in task-related functional connectivity within the fronto-temporal language network, and significant modulation in MEG indices of auditory/language processing in the direction of more efficient processing.

## 4 Discussion

Both animal model and human studies implicate glutamatergic and GABAergic mechanisms in the etiology of autism spectrum disorders. Additionally, multiple pharmacological agents acting on these systems (e.g., arbaclofen and d-cyclosterine) are currently in clinical trials for the treatment of core and associated symptoms of ASD (140, 141). However, links between aberrant levels of GABA and glutamate with network-wide dysfunction or clinical symptom presentation/severity need to be further characterized and understood within a nomological network to aid in developing pharmacological and non-pharmacological treatments. Furthermore, there is limited understanding of how acute or long-term modulation in the concentrations of these neurotransmitters affects brain network functioning or behavioral symptom presentation in a child, adolescent, or adult. Repetitive Transcranial Magnetic Stimulation has the capacity to modulate functional brain networks. cTBS specifically appears to exert its effect via glutamatergic and GABAergic mechanisms (66–69). Additionally, the strong safety record of rTMS and FDA approval for other neuropsychiatric disorders makes it a promising tool to conduct both the current proof of mechanism study and potential future clinical trials.

The present study is the first to assess the impact of a single administration of cTBS on auditory/language processing in adolescents with ASD. It is anticipated that this study will inform the degree to which an imbalance in glutamate/GABA ratio contributes to dysfunction at the structural and functional network level and to ASD symptom presentation especially as it relates to auditory and language processing and how acute targeted modulation at the molecular and functional network level impacts language processing. The advent of advanced neuroimaging and neurostimulation tools has allowed us, in recent years, to conduct such translational, experimental therapeutics study designs. If successful, this approach may: 1. Provide valuable data on the safety, feasibility, and tolerability of cTBS to this cortical region in a sample of adolescents with ASD; 2. Identify diagnostic and/or predictive biomarkers of response to treatment; and 3. Provide an explanatory neural mechanism supporting future clinical trials using cTBS to improve language processing in adolescents with ASD. Currently, we have begun recruitment and data collection for the ASD phase of the study. While recruitment for the pilot phase of the study was impacted by the COVID19 pandemic, we anticipate recruitment of the main phase (86 adolescents with ASD) to be completed by Summer, 2025. Quality control and preprocessing will be ongoing throughout data collection to ensure data pipelines are robust.

Study design limitations were recognized and addressed between phases. One limitation immediately recognized was that the length of a visit or number of visits could be burdensome to the participant (fatigue) and the family (scheduling). We address this limitation by offering flexibility to the participants and families to split (decrease fatigue) or combine (reduce scheduling conflicts) the screening and baseline visits and offer a flexible schedule including evening and weekend visits. An anticipated limitation was motion within the imaging scanners. MRI is sensitive to motion, MEG less so. Therefore, to aid in motion reduction we added practice in a mock scanner prior to MRI data collection. The mock scanner produces noises similar to the MRI scan sequences that will be acquired, allowing the participant to familiarize themselves with the sounds of the scanner. In addition, during the mock scanner session, the participant has a tracker attached to their forehead that measures motion and provides visual feedback of their movement. The participant can practice until movement is minimized. Lastly, we recognize that our study procedures may limit to those not on certain exclusionary medications (e.g., GABAergic medications or those that might decrease seizure threshold), those without co-occurring intellectual disability, those without a history of seizures, those without severe sensory sensitivities, and those able to tolerate remaining still for an extended amount of time. Additionally, the study age range is currently limited to 14-17 year olds. We recognize that this represents a small subgroup of those with ASD and that these inclusion/exclusion criteria, which were designed to optimize the safety, feasibility, and tolerability of study procedures may limit the generalizability of our findings beyond this specific subgroup of individuals with ASD. We also decided not to limit our inclusion criteria to those with significant language impairments. As the main aim of the study was to relate measures of local E/I balance to measures of network connectivity and ultimately to ASD symptoms, it was determined that statistically, we will benefit from having a range of behavioral symptom severity in the auditory/language processing domain. Additionally, as we observed relationships between these variables and induced a change from pre-post cTBS in the pilot (Healthy Volunteer) sample, we believe it will be present, even if the person does not show clinically significant symptoms. That being said, the observed relationships and/or effects of the cTBS may be more profound in those with more severe symptoms and future studies may probe these outcomes in those with Specific Language Impairment and/or Semantic-Pragmatic Disorder.

This study was designed as a proof-of-mechanism study and is not dosed nor powered to induce long-term clinical effects. However, if this study finds that cTBS to left pSTC leads to acutely reduced glutamate/GABA ratio, increased functional connectivity within the language network, reduced latency of the M100/M50, or enhanced induced gamma power on the MEG, this would suggest that if multiple sessions were applied, one may see improvements in auditory (both basic and language) processing and potentially improvements in speech and/or theory of mind and other pragmatic/social language comprehension skills through networks connecting the left pSTC to inferior frontal and right pSTC regions. However, multiple sessions would need to be applied before one might expect to see behavioral changes in these domains. These studies have yet to be conducted.

If we do not see a baseline relationship or post cTBS change in our hypothesized outcomes, we will explore alternative theories implicating other pathophysiological mechanisms other than E/I imbalance or network connectivity that may better explain our findings. Additionally, if we find that our prespecified networks or analyses do not adequately capture the variability in response, alternative outcomes (e.g. trial by trial variability in evoked responses to both the fMRI and MEG tasks or behavioral reaction time to the ADDT task) or processing strategies (e.g. ICA-defined functional networks) may be explored. De-identified raw data from this project will be made publicly available on all data from participants who consent to this type of data sharing, allowing for data sharing with other research groups who may be interested in applying other analytic approaches to the data. Future studies will also need to be conducted to evaluate factors that may contribute to the efficacy and durability of a clinical intervention effect. Specifically, factors shown to impact the clinical efficacy of other rTMS protocols which include the number of sessions (142), target and targeting strategy (143, 144), stimulation intensity, frequency of stimulation and frequency of sessions (145, 146), and whether adjunctive or concurrent behavioral or physiological tasks or interventions are applied (147).

There are relatively few evidence-based options for the treatment of the core symptoms of ASD. If our study indicates that cTBS can modulate glutamate/GABA ratios and this modulation is associated with improvements in MEG indices of auditory/language processing, it will provide the rationale to explore this as a potential novel therapeutic intervention for specific impairments associated with ASD.

## Data availability statement

The raw data supporting the conclusions of this article will be made available by the authors, without undue reservation.

## Ethics statement

The studies involving humans were approved by NIH Institutional Review Board. The studies were conducted in accordance with the local legislation and institutional requirements. Written informed consent for participation in this study was provided by the participants’ legal guardians/next of kin.

## Author contributions

LO: Conceptualization, Data curation, Formal analysis, Investigation, Methodology, Project administration, Supervision, Validation, Writing – original draft, Writing – review & editing. SF: Data curation, Formal analysis, Investigation, Methodology, Validation, Writing – original draft, Writing – review & editing. LB: Data curation, Formal analysis, Methodology, Software, Validation, Writing – original draft, Writing – review & editing. MH: Data curation, Formal analysis, Investigation, Software, Writing – original draft, Writing – review & editing. MJ: Data curation, Formal analysis, Investigation, Software, Visualization, Writing – original draft, Writing – review & editing. PR: Data curation, Formal analysis, Software, Visualization, Writing – original draft, Writing – review & editing. Z-DD: Data curation, Formal analysis, Methodology, Software, Writing – original draft, Writing – review & editing. JS: Data curation, Formal analysis, Methodology, Software, Writing – original draft, Writing – review & editing. JV: Data curation, Formal analysis, Methodology, Software, Writing – original draft, Writing – review & editing. SL: Methodology, Resources, Supervision, Writing – review & editing, Conceptualization, Funding acquisition.

## References

[1] APA . Diagnostic and statistical manual of mental disorders. 5 ed. Arlington, VA: American Psychiatric Publishing (2013).

[2] Kanner L . Autistic disturbances of affective contact. Nervous Child (1943) 2:217–50. 4880460

[3] Adamek J Luo Y Ewen J . Using connectivity to explain neuropsychiatric conditions: The example of autism. In: Thakor NV , editor. Handbook of neuroengineering. Singapore: Springer (2022).

[4] Ewen J . Psychological theories of autism: everything, all at once, in one place. PsyArXiv (2023). doi: 10.31234/osf.io/kguq2

[5] Rubenstein JL Merzenich MM . Model of autism: increased ratio of excitation/inhibition in key neural systems. Genes Brain Behav (2003) 2(5):255–67. doi: 10.1034/j.1601-183X.2003.00037.x PMC6748642 14606691

[6] Sears SM Hewett SJ . Influence of glutamate and GABA transport on brain excitatory/inhibitory balance. Exp Biol Med (Maywood) (2021) 246(9):1069–83. doi: 10.1177/1535370221989263 PMC8113735 33554649

[7] Blatt GJ Fatemi SH . Alterations in GABAergic biomarkers in the autism brain: research findings and clinical implications. Anat Rec (Hoboken) (2011) 294(10):1646–52. doi: 10.1002/ar.21252 PMC3190183 21901839

[8] Montanari M Martella G Bonsi P Meringolo M . Autism spectrum disorder: focus on glutamatergic neurotransmission. Int J Mol Sci (2022) 23(7):3861. doi: 10.3390/ijms23073861 35409220 PMC8998955

[9] Oberman LM Rotenberg A Pascual-Leone A . Aberrant brain plasticity in autism spectrum disorders. In: Tracy JI Hampstead BM Sathian K , editors. Cognitive plasticity in neurological disorders. New York: Oxford University Press (2015).

[10] Belmonte MK Allen G Beckel-Mitchener A Boulanger LM Carper RA Webb SJ . Autism and abnormal development of brain connectivity. J Neurosci (2004) 24(42):9228–31. doi: 10.1523/JNEUROSCI.3340-04.2004 PMC6730085 15496656

[11] Belmonte MK Cook EH Jr. Anderson GM Rubenstein JL Greenough WT Beckel-Mitchener A . Autism as a disorder of neural information processing: directions for research and targets for therapy. Mol Psychiatry (2004) 9(7):646–63. doi: 10.1038/sj.mp.4001499 15037868

[12] Shukla DK Keehn B Smylie DM Muller RA . Microstructural abnormalities of short-distance white matter tracts in autism spectrum disorder. Neuropsychologia (2011) 49(5):1378–82. doi: 10.1016/j.neuropsychologia.2011.02.022 PMC3482113 21333661

[13] Fletcher PT Whitaker RT Tao R DuBray MB Froehlich A Ravichandran C . Microstructural connectivity of the arcuate fasciculus in adolescents with high-functioning autism. Neuroimage (2010) 51(3):1117–25. doi: 10.1016/j.neuroimage.2010.01.083 PMC2966943 20132894

[14] Cheung C Chua SE Cheung V Khong PL Tai KS Wong TK . White matter fractional anisotrophy differences and correlates of diagnostic symptoms in autism. J Child Psychol Psychiatry (2009) 50(9):1102–12. doi: 10.1111/j.1469-7610.2009.02086.x 19490309

[15] Larson C Thomas HR Crutcher J Stevens MC Eigsti I-M . Language Networks in Autism Spectrum Disorder: A systematic review of connectivity-based fMRI studies. Rev J Autism Dev Disord (2023), 1–28. doi: 10.1007/s40489-023-00382-6 37363697

[16] Dinstein I Heeger DJ Lorenzi L Minshew NJ Malach R Behrmann M . Unreliable evoked responses in autism. Neuron (2012) 75(6):981–91. doi: 10.1016/j.neuron.2012.07.026 PMC3457023 22998867

[17] Simmons DR Robertson AE McKay LS Toal E McAleer P Pollick FE . Vision in autism spectrum disorders. Vision Res (2009) 49(22):2705–39. doi: 10.1016/j.visres.2009.08.005 19682485

[18] Simmons D Milne E . Response to Davis and Plaisted-Grant: low or high endogenous neural noise in autism spectrum disorder? Autism (2015) 19(3):363–4. doi: 10.1177/1362361314557683 25488003

[19] Davis G Plaisted-Grant K . Low endogenous neural noise in autism. Autism (2015) 19(3):351–62. doi: 10.1177/1362361314552198 25248666

[20] Tomasi D Volkow ND . Reduced local and increased long-range functional connectivity of the thalamus in autism spectrum disorder. Cereb Cortex (2019) 29(2):573–85. doi: 10.1093/cercor/bhx340 PMC6319176 29300843

[21] Butler JS Molholm S Andrade GN Foxe JJ . An examination of the neural unreliability thesis of autism. Cereb Cortex (2017) 27(1):185–200. doi: 10.1093/cercor/bhw375 27923839 PMC5939224

[22] Beauchamp MS Yasar NE Frye RE Ro T . Touch, sound and vision in human superior temporal sulcus. Neuroimage (2008) 41(3):1011–20. doi: 10.1016/j.neuroimage.2008.03.015 PMC2409200 18440831

[23] Gordon I Voos AC Bennett RH Bolling DZ Pelphrey KA Kaiser MD . Brain mechanisms for processing affective touch. Hum Brain Mapp (2013) 34(4):914–22. doi: 10.1002/hbm.21480 PMC6869848 22125232

[24] Hagan CC Woods W Johnson S Calder AJ Green GG Young AW . MEG demonstrates a supra-additive response to facial and vocal emotion in the right superior temporal sulcus. Proc Natl Acad Sci USA (2009) 106(47):20010–5. doi: 10.1073/pnas.0905792106 PMC2785283 19906999

[25] Hagan CC Woods W Johnson S Green GG Young AW . Involvement of right STS in audio-visual integration for affective speech demonstrated using MEG. PloS One (2013) 8(8):e70648. doi: 10.1371/journal.pone.0070648 23950977 PMC3741276

[26] Hocking J Price CJ . The role of the posterior superior temporal sulcus in audiovisual processing. Cereb Cortex (2008) 18(10):2439–49. doi: 10.1093/cercor/bhn007 PMC2536697 18281303

[27] Vasileiadi M Schuler AL Woletz M Linhardt D Windischberger C Tik M . Functional connectivity explains how neuronavigated TMS of posterior temporal subregions differentially affect language processing. Brain Stimul (2023) 16(4):1062–71. doi: 10.1016/j.brs.2023.06.014 37390891

[28] Kanner L . Irrelevant and metaphorical language in early infantile autism. Am J Psychiatry (1946) 103(2):242–6. doi: 10.1176/ajp.103.2.242 21001998

[29] Goncalves AM Monteiro P . Autism Spectrum Disorder and auditory sensory alterations: a systematic review on the integrity of cognitive and neuronal functions related to auditory processing. J Neural Transm (Vienna) (2023) 130(3):325–408. doi: 10.1007/s00702-023-02595-9 36914900 PMC10033482

[30] Tang B Levine M Adamek JH Wodka EL Caffo BS Ewen JB . Evaluating causal psychological models: A study of language theories of autism using a large sample. Front Psychol (2023) 14:1060525. doi: 10.3389/fpsyg.2023.1060525 36910768 PMC9998497

[31] Tanigawa J Kagitani-Shimono K Matsuzaki J Ogawa R Hanaie R Yamamoto T . Atypical auditory language processing in adolescents with autism spectrum disorder. Clin Neurophysiol (2018) 129(9):2029–37. doi: 10.1016/j.clinph.2018.05.014 29934264

[32] Yau SH Brock J McArthur G . The relationship between spoken language and speech and nonspeech processing in children with autism: a magnetic event-related field study. Dev Sci (2016) 19(5):834–52. doi: 10.1111/desc.12328 27146167

[33] Williams BT Gray KM . The relationship between emotion recognition ability and social skills in young children with autism. Autism (2013) 17(6):762–8. doi: 10.1177/1362361312465355 23175751

[34] Wang AT Lee SS Sigman M Dapretto M . Neural basis of irony comprehension in children with autism: the role of prosody and context. Brain (2006) 129(Pt 4):932–43. doi: 10.1093/brain/awl032 PMC3713234 16481375

[35] Tesink CM Buitelaar JK Petersson KM van der Gaag RJ Kan CC Tendolkar I . Neural correlates of pragmatic language comprehension in autism spectrum disorders. Brain (2009) 132(Pt 7):1941–52. doi: 10.1093/brain/awp103 19423680

[36] Mason RA Williams DL Kana RK Minshew N Just MA . Theory of Mind disruption and recruitment of the right hemisphere during narrative comprehension in autism. Neuropsychologia (2008) 46(1):269–80. doi: 10.1016/j.neuropsychologia.2007.07.018 PMC2259382 17869314

[37] Knaus TA Silver AM Lindgren KA Hadjikhani N Tager-Flusberg H . fMRI activation during a language task in adolescents with ASD. J Int Neuropsychol Soc (2008) 14(6):967–79. doi: 10.1017/S1355617708081216 PMC2747321 18954477

[38] Just MA Cherkassky VL Keller TA Minshew NJ . Cortical activation and synchronization during sentence comprehension in high-functioning autism: evidence of underconnectivity. Brain (2004) 127(Pt 8):1811–21. doi: 10.1093/brain/awh199 15215213

[39] Saygin AP Dick F Wilson SM Dronkers NF Bates E . Neural resources for processing language and environmental sounds: evidence from aphasia. Brain (2003) 126(Pt 4):928–45. doi: 10.1093/brain/awg082 12615649

[40] Duffy FH Eksioglu YZ Rotenberg A Madsen JR Shankardass A Als H . The frequency modulated auditory evoked response (FMAER), a technical advance for study of childhood language disorders: cortical source localization and selected case studies. BMC Neurol (2013) 13:12. doi: 10.1186/1471-2377-13-12 23351174 PMC3582442

[41] Williams DL Cherkassky VL Mason RA Keller TA Minshew NJ Just MA . Brain function differences in language processing in children and adults with autism. Autism Res (2013) 6(4):288–302. doi: 10.1002/aur.1291 23495230 PMC4492467

[42] Park KY Lee JJ Dierker D Marple LM Hacker CD Roland JL . Mapping language function with task-based vs. resting-state functional MRI. PloS One (2020) 15(7):e0236423. doi: 10.1371/journal.pone.0236423 32735611 PMC7394427

[43] Castro AC Monteiro P . Auditory dysfunction in animal models of autism spectrum disorder. Front Mol Neurosci (2022) 15:845155. doi: 10.3389/fnmol.2022.845155 35493332 PMC9043325

[44] Choi IY Andronesi OC Barker P Bogner W Edden RAE Kaiser LG . Spectral editing in (1) H magnetic resonance spectroscopy: Experts' consensus recommendations. NMR Biomed (2021) 34(5):e4411. doi: 10.1002/nbm.4411 32946145 PMC8557623

[45] Geramita M van der Veen JW Barnett AS Savostyanova AA Shen J Weinberger DR . Reproducibility of prefrontal gamma-aminobutyric acid measurements with J-edited spectroscopy. NMR Biomed (2011) 24(9):1089–98. doi: 10.1002/nbm.1662 21290458

[46] Castellanos FX Cortese S Proal E . Connectivity. Curr Top Behav Neurosci (2014) 16:49–77. doi: 10.1007/978-3-662-45758-0_244 23943564

[47] Khan S Gramfort A Shetty NR Kitzbichler MG Ganesan S Moran JM . Local and long-range functional connectivity is reduced in concert in autism spectrum disorders. Proc Natl Acad Sci USA (2013) 110(8):3107–12. doi: 10.1073/pnas.1214533110 PMC3581984 23319621

[48] Berman JI Liu S Bloy L Blaskey L Roberts TP Edgar JC . Alpha-to-gamma phase-amplitude coupling methods and application to autism spectrum disorder. Brain Connect (2015) 5(2):80–90. doi: 10.1089/brain.2014.0242 25109843 PMC4361390

[49] Berman JI Edgar JC Blaskey L Kuschner ES Levy SE Ku M . Multimodal diffusion-MRI and MEG assessment of auditory and language system development in autism spectrum disorder. Front Neuroanat (2016) 10:30. doi: 10.3389/fnana.2016.00030 27047349 PMC4803725

[50] Roberts TP Heiken K Kahn SY Qasmieh S Blaskey L Solot C . Delayed magnetic mismatch negativity field, but not auditory M100 response, in specific language impairment. Neuroreport (2012) 23(8):463–8. doi: 10.1097/WNR.0b013e32835202b6 PMC3345126 22551948

[51] Port RG Edgar JC Ku M Bloy L Murray R Blaskey L . Maturation of auditory neural processes in autism spectrum disorder - A longitudinal MEG study. NeuroImage Clin (2016) 11:566–77. doi: 10.1016/j.nicl.2016.03.021 PMC4844592 27158589

[52] Port RG Anwar AR Ku M Carlson GC Siegel SJ Roberts TP . Prospective MEG biomarkers in ASD: pre-clinical evidence and clinical promise of electrophysiological signatures. Yale J Biol Med (2015) 88(1):25–36. 25745372 PMC4345535

[53] Roberts TPL Kuschner ES Edgar JC . Biomarkers for autism spectrum disorder: opportunities for magnetoencephalography (MEG). J Neurodev Disord (2021) 13(1):34. doi: 10.1186/s11689-021-09385-y 34525943 PMC8442415

[54] Gandal MJ Edgar JC Ehrlichman RS Mehta M Roberts TP Siegel SJ . Validating gamma oscillations and delayed auditory responses as translational biomarkers of autism. Biol Psychiatry (2010) 68(12):1100–6. doi: 10.1016/j.biopsych.2010.09.031 PMC5070466 21130222

[55] Snijders TM Milivojevic B Kemner C . Atypical excitation-inhibition balance in autism captured by the gamma response to contextual modulation. NeuroImage Clin (2013) 3:65–72. doi: 10.1016/j.nicl.2013.06.015 24179850 PMC3791282

[56] Balz J Keil J Roa Romero Y Mekle R Schubert F Aydin S . GABA concentration in superior temporal sulcus predicts gamma power and perception in the sound-induced flash illusion. Neuroimage (2016) 125:724–30. doi: 10.1016/j.neuroimage.2015.10.087 26546865

[57] Port RG Gaetz W Bloy L Wang DJ Blaskey L Kuschner ES . Exploring the relationship between cortical GABA concentrations, auditory gamma-band responses and development in ASD: Evidence for an altered maturational trajectory in ASD. Autism Res (2017) 10(4):593–607. doi: 10.1002/aur.1686 27696740 PMC5376374

[58] Kahkonen S Marttinen Rossi E Yamashita H . Alcohol impairs auditory processing of frequency changes and novel sounds: a combined MEG and EEG study. Psychopharmacol (Berl) (2005) 177(4):366–72. doi: 10.1007/s00213-004-1960-1 15290001

[59] Jensen O Mazaheri A . Shaping functional architecture by oscillatory alpha activity: gating by inhibition. Front Hum Neurosci (2010) 4:186. doi: 10.3389/fnhum.2010.00186 21119777 PMC2990626

[60] Klimesch W Sauseng P Hanslmayr S . EEG alpha oscillations: the inhibition-timing hypothesis. Brain Res Rev (2007) 53(1):63–88. doi: 10.1016/j.brainresrev.2006.06.003 16887192

[61] Muthukumaraswamy SD Edden RA Jones DK Swettenham JB Singh KD . Resting GABA concentration predicts peak gamma frequency and fMRI amplitude in response to visual stimulation in humans. Proc Natl Acad Sci USA (2009) 106(20):8356–61. doi: 10.1073/pnas.0900728106 PMC2688873 19416820

[62] Edden RA Muthukumaraswamy SD Freeman TC Singh KD . Orientation discrimination performance is predicted by GABA concentration and gamma oscillation frequency in human primary visual cortex. J Neurosci (2009) 29(50):15721–6. doi: 10.1523/JNEUROSCI.4426-09.2009 PMC6666191 20016087

[63] Edgar JC Khan SY Blaskey L Chow VY Rey M Gaetz W . Neuromagnetic oscillations predict evoked-response latency delays and core language deficits in autism spectrum disorders. J Autism Dev Disord (2015) 45(2):395–405. doi: 10.1007/s10803-013-1904-x 23963591 PMC5012005

[64] Huang YZ Edwards MJ Rounis E Bhatia KP Rothwell JC . Theta burst stimulation of the human motor cortex. Neuron (2005) 45(2):201–6. doi: 10.1016/j.neuron.2004.12.033 15664172

[65] Oberman L Eldaief M Fecteau S Ifert-Miller F Tormos JM Pascual-Leone A . Abnormal modulation of corticospinal excitability in adults with Asperger's syndrome. Eur J Neurosci (2012) 36(6):2782–8. doi: 10.1111/j.1460-9568.2012.08172.x PMC3524970 22738084

[66] Benali A Trippe J Weiler E Mix A Petrasch-Parwez E Girzalsky W . Theta-burst transcranial magnetic stimulation alters cortical inhibition. J Neurosci (2011) 31(4):1193–203. doi: 10.1523/JNEUROSCI.1379-10.2011 PMC6623597 21273404

[67] Stagg CJ Wylezinska M Matthews PM Johansen-Berg H Jezzard P Rothwell JC . Neurochemical effects of theta burst stimulation as assessed by magnetic resonance spectroscopy. J Neurophysiol (2009) 101(6):2872–7. doi: 10.1152/jn.91060.2008 PMC2694115 19339458

[68] Huang YZ Chen RS Rothwell JC Wen HY . The after-effect of human theta burst stimulation is NMDA receptor dependent. Clin Neurophysiol (2007) 118(5):1028–32. doi: 10.1016/j.clinph.2007.01.021 17368094

[69] Cember ATJ Deck BL Kelkar A Faseyitan O Zimmerman JP Erickson B . Glutamate-weighted magnetic resonance imaging (GluCEST) detects effects of transcranial magnetic stimulation to the motor cortex. Neuroimage (2022) 256:119191. doi: 10.1016/j.neuroimage.2022.119191 35413447

[70] Solomon EA Sperling MR Sharan AD Wanda PA Levy DF Lyalenko A . Theta-burst stimulation entrains frequency-specific oscillatory responses. Brain Stimul (2021) 14(5):1271–84. doi: 10.1016/j.brs.2021.08.014 PMC9161680 34428553

[71] Thut G Veniero D Romei V Miniussi C Schyns P Gross J . Rhythmic TMS causes local entrainment of natural oscillatory signatures. Curr Biol (2011) 21(14):1176–85. doi: 10.1016/j.cub.2011.05.049 PMC3176892 21723129

[72] Schwarzkopf DS Silvanto J Rees G . Stochastic resonance effects reveal the neural mechanisms of transcranial magnetic stimulation. J Neurosci (2011) 31(9):3143–7. doi: 10.1523/JNEUROSCI.4863-10.2011 PMC3059801 21368025

[73] Moss F Ward LM Sannita WG . Stochastic resonance and sensory information processing: a tutorial and review of application. Clin Neurophysiol (2004) 115(2):267–81. doi: 10.1016/j.clinph.2003.09.014 14744566

[74] Tanaka K Kawakatsu M Nemoto I . Stochastic resonance seen as increase in phase synchrony or power in auditory steady-state responses in MEG. Annu Int Conf IEEE Eng Med Biol Soc (2007) 2007:2500–3. doi: 10.1109/IEMBS.2007.4352836 18002502

[75] Tanaka K Kawakatsu M Nemoto I . Stochastic resonance in auditory steady-state responses in a magnetoencephalogram. Clin Neurophysiol (2008) 119(9):2104–10. doi: 10.1016/j.clinph.2008.05.007 18621581

[76] Ni HC Lin HY Chen YL Hung J Wu CT Wu YY . 5-day multi-session intermittent theta burst stimulation over bilateral posterior superior temporal sulci in adults with autism-a pilot study. BioMed J (2022) 45(4):696–707. doi: 10.1016/j.bj.2021.07.008 34358713 PMC9486126

[77] Ni HC Chen YL Chao YP Wu CT Wu YY Liang SH . Intermittent theta burst stimulation over the posterior superior temporal sulcus for children with autism spectrum disorder: A 4-week randomized blinded controlled trial followed by another 4-week open-label intervention. Autism (2021) 25(5):1279–94. doi: 10.1177/1362361321990534 33631943

[78] Ni HC Chao YP Tseng RY Wu CT Cocchi L Chou TL . Lack of effects of four-week theta burst stimulation on white matter macro/microstructure in children and adolescents with autism. NeuroImage Clin (2023) 37:103324. doi: 10.1016/j.nicl.2023.103324 36638598 PMC9852693

[79] Ni HC Chen YL Chao YP Wu CT Chen RS Chou TL . A lack of efficacy of continuous theta burst stimulation over the left dorsolateral prefrontal cortex in autism: A double blind randomized sham-controlled trial. Autism Res (2023) 16(6):1247–62. doi: 10.1002/aur.2954 37219040

[80] Cardin JA Carlen M Meletis K Knoblich U Zhang F Deisseroth K . Driving fast-spiking cells induces gamma rhythm and controls sensory responses. Nature (2009) 459(7247):663–7. doi: 10.1038/nature08002 PMC3655711 19396156

[81] Lehr R . Sixteen S-squared over D-squared: a relation for crude sample size estimates. Stat Med (1992) 11(8):1099–102. doi: 10.1002/sim.4780110811 1496197

[82] Cohen J . Statistical power analysis for the behavioral sciences. New York, NY: Routledge Academic (1988).

[83] Constantino JN Gruber CP . Social responsiveness scale. 2nd ed. Los Angeles: Western Psychological Services (2012).

[84] Achenbach TM Rescorla LA . Manual for the ASEBA school-age forms and profiles. Burlington, VT: University of Vermont, Research Center for Children, Youth, and Families (2001).

[85] Wechsler D Zhou X . WASI-II: WEchsler abbreviated scale of intelligence. 2nd ed. San Antonio: Psychological Corporation (2011).

[86] Sparrow SS Cicchetti DV Balla DA . Vineland-3: Vineland adaptive behavior scales. Manual. Minneapolis: Pearson Assessments (2016).

[87] Lord C Rutter M Le Couteur A . Autism Diagnostic Interview-Revised: a revised version of a diagnostic interview for caregivers of individuals with possible pervasive developmental disorders. J Autism Dev Disord (1994) 24(5):659–85. doi: 10.1007/BF02172145 7814313

[88] Williams KT . EVT-2: expressive vocabulary test. 2nd ed. San Antonio: Pearson (2007).

[89] Dunn LM Dunn DM . PPVT-4: Peabody picture vocabulary test. 4th ed. Minneapolis: Pearson Assessments (2007).

[90] Wiig EH Semel EM Secord W . CELF-5: clinical evaluation of language fundamentals. 5th ed. Bloomington: Pearson (2013).

[91] Roberts TP Khan SY Rey M Monroe JF Cannon K Blaskey L . MEG detection of delayed auditory evoked responses in autism spectrum disorders: towards an imaging biomarker for autism. Autism Res (2010) 3(1):8–18. doi: 10.1002/aur.111 20063319 PMC3099241

[92] Pantev C Bertrand O Eulitz C Verkindt C Hampson S Schuierer G . Specific tonotopic organizations of different areas of the human auditory cortex revealed by simultaneous magnetic and electric recordings. Electroencephalogr Clin Neurophysiol (1995) 94(1):26–40. doi: 10.1016/0013-4694(94)00209-4 7530637

[93] Yvert B Crouzeix A Bertrand O Seither-Preisler A Pantev C . Multiple supratemporal sources of magnetic and electric auditory evoked middle latency components in humans. Cereb Cortex (2001) 11(5):411–23. doi: 10.1093/cercor/11.5.411 11313293

[94] Oram Cardy JE Ferrari P Flagg EJ Roberts W Roberts TP . Prominence of M50 auditory evoked response over M100 in childhood and autism. Neuroreport (2004) 15(12):1867–70. doi: 10.1097/00001756-200408260-00006 15305126

[95] Edgar JC Franzen RE McNamee M Green HL Shen G DiPiero M . A comparison of resting-state eyes-closed and dark-room alpha-band activity in children. Psychophysiology (2023) 60(6):e14285. doi: 10.1111/psyp.14285 36929476 PMC11097109

[96] Roberts TP Cannon KM Tavabi K Blaskey L Khan SY Monroe JF . Auditory magnetic mismatch field latency: a biomarker for language impairment in autism. Biol Psychiatry (2011) 70(3):263–9. doi: 10.1016/j.biopsych.2011.01.015 PMC3134608 21392733

[97] Deng ZD Lisanby SH Peterchev AV . Electric field depth-focality tradeoff in transcranial magnetic stimulation: simulation comparison of 50 coil designs. Brain Stimul (2013) 6(1):1–13. doi: 10.1016/j.brs.2012.02.005 22483681 PMC3568257

[98] Gaillard WD Berl MM Moore EN Ritzl EK Rosenberger LR Weinstein SL . Atypical language in lesional and nonlesional complex partial epilepsy. Neurology (2007) 69(18):1761–71. doi: 10.1212/01.wnl.0000289650.48830.1a 17967992

[99] Berl MM Zimmaro LA Khan OI Dustin I Ritzl E Duke ES . Characterization of atypical language activation patterns in focal epilepsy. Ann Neurol (2014) 75(1):33–42. doi: 10.1002/ana.24015 24038442 PMC4209919

[100] You X Adjouadi M Guillen MR Ayala M Barreto A Rishe N . Sub-patterns of language network reorganization in pediatric localization related epilepsy: a multisite study. Hum Brain Mapp (2011) 32(5):784–99. doi: 10.1002/hbm.21066 PMC3640868 21484949

[101] Austermuehle A Cocjin J Reynolds R Agrawal S Sepeta L Gaillard WD . Language functional MRI and direct cortical stimulation in epilepsy preoperative planning. Ann Neurol (2017) 81(4):526–37. doi: 10.1002/ana.24899 PMC5401636 28220524

[102] Xiong J Rao S Jerabek P Zamarripa F Woldorff M Lancaster J . Intersubject variability in cortical activations during a complex language task. Neuroimage (2000) 12(3):326–39. doi: 10.1006/nimg.2000.0621 10944415

[103] Mesulam MM Thompson CK Weintraub S Rogalski EJ . The Wernicke conundrum and the anatomy of language comprehension in primary progressive aphasia. Brain (2015) 138(Pt 8):2423–37. doi: 10.1093/brain/awv154 PMC4805066 26112340

[104] Rosenberger LR Zeck J Berl MM Moore EN Ritzl EK Shamim S . Interhemispheric and intrahemispheric language reorganization in complex partial epilepsy. Neurology (2009) 72(21):1830–6. doi: 10.1212/WNL.0b013e3181a7114b PMC2690987 19470965

[105] Oberman LM Ifert-Miller F Najib U Bashir S Heydrich JG Picker J . Abnormal mechanisms of plasticity and metaplasticity in autism spectrum disorders and fragile X syndrome. J Child Adolesc Psychopharmacol (2016) 26(7):617–24. doi: 10.1089/cap.2015.0166 PMC5111832 27218148

[106] Oberman LM Pascual-Leone A Rotenberg A . Modulation of corticospinal excitability by transcranial magnetic stimulation in children and adolescents with autism spectrum disorder. Front Hum Neurosci (2014) 8:627. doi: 10.3389/fnhum.2014.00627 25165441 PMC4131188

[107] Oberman L Ifert-Miller F Najib U Bashir S Woollacott I Gonzalez-Heydrich J . Transcranial magnetic stimulation provides means to assess cortical plasticity and excitability in humans with fragile x syndrome and autism spectrum disorder. Front Synaptic Neurosci (2010) 2:26. doi: 10.3389/fnsyn.2010.00026 21423512 PMC3059673

[108] Niso G Gorgolewski KJ Bock E Brooks TL Flandin G Gramfort A . MEG-BIDS, the brain imaging data structure extended to magnetoencephalography. Sci Data (2018) 5:180110. doi: 10.1038/sdata.2018.110 29917016 PMC6007085

[109] Appelhoff S Sanderson M Brooks TL van Vliet M Quentin R Holdgraf C . MNE-BIDS: Organizing electrophysiological data into the BIDS format and facilitating their analysis. J Open Source Softw (2019) 4(44):1896. doi: 10.21105/joss.01896 35990374 PMC9390980

[110] Gramfort A Luessi M Larson E Engemann DA Strohmeier D Brodbeck C . MNE software for processing MEG and EEG data. Neuroimage (2014) 86:446–60. doi: 10.1016/j.neuroimage.2013.10.027 PMC3930851 24161808

[111] Dale AM Fischl B Sereno MI . Cortical surface-based analysis. I. Segmentation and surface reconstruction. Neuroimage (1999) 9(2):179–94. doi: 10.1006/nimg.1998.0395 9931268

[112] Edgar JC Fisk C Liu S Pandey J Herrington JD Schultz RT . Translating adult electrophysiology findings to younger patient populations: difficulty measuring 40-hz auditory steady-state responses in typically developing children and children with autism spectrum disorder. Dev Neurosci (2016) 38(1):1–14. doi: 10.1159/000441943 26730806 PMC4732910

[113] van der Veen JW Marenco S Berman KF Shen J . Retrospective correction of frequency drift in spectral editing: The GABA editing example. NMR Biomed (2017) 30(8). doi: 10.1002/nbm.3725 PMC5511084 28370463

[114] Tournier JD Smith R Raffelt D Tabbara R Dhollander T Pietsch M . MRtrix3: A fast, flexible and open software framework for medical image processing and visualisation. Neuroimage (2019) 202:116137. doi: 10.1016/j.neuroimage.2019.116137 31473352

[115] Jenkinson M Beckmann CF Behrens TE Woolrich MW Smith SM . Fsl. Neuroimage (2012) 62(2):782–90. doi: 10.1016/j.neuroimage.2011.09.015 21979382

[116] Fischl B . FreeSurfer. Neuroimage (2012) 62(2):774–81. doi: 10.1016/j.neuroimage.2012.01.021 PMC3685476 22248573

[117] Avants BB Tustison N Song G . Advanced normalization tools (ANTS). Insight J (2009) 2(365):1–35. doi: 10.54294/uvnhin

[118] Wasserthal J Neher P Maier-Hein KH . TractSeg - Fast and accurate white matter tract segmentation. Neuroimage (2018) 183:239–53. doi: 10.1016/j.neuroimage.2018.07.070 30086412

[119] Warrington S Bryant KL Khrapitchev AA Sallet J Charquero-Ballester M Douaud G . XTRACT - Standardised protocols for automated tractography in the human and macaque brain. Neuroimage (2020) 217:116923. doi: 10.1016/j.neuroimage.2020.116923 32407993 PMC7260058

[120] Cox RW . AFNI: software for analysis and visualization of functional magnetic resonance neuroimages. Comput BioMed Res (1996) 29(3):162–73. doi: 10.1006/cbmr.1996.0014 8812068

[121] Reynolds RC Taylor PA Glen DR . Quality control practices in FMRI analysis: Philosophy, methods and examples using AFNI. Front Neurosci (2022) 16:1073800. doi: 10.3389/fnins.2022.1073800 36793774 PMC9922690

[122] Saad ZS Glen DR Chen G Beauchamp MS Desai R Cox RW . A new method for improving functional-to-structural MRI alignment using local Pearson correlation. Neuroimage (2009) 44(3):839–48. doi: 10.1016/j.neuroimage.2008.09.037 PMC2649831 18976717

[123] Glen DR Taylor PA Buchsbaum BR Cox RW Reynolds RC . Beware (Surprisingly common) left-right flips in your MRI data: an efficient and robust method to check MRI dataset consistency using AFNI. Front Neuroinform (2020) 14:18. doi: 10.3389/fninf.2020.00018 32528270 PMC7263312

[124] Thielscher A Antunes A Saturnino GB . Field modeling for transcranial magnetic stimulation: A useful tool to understand the physiological effects of TMS? Annu Int Conf IEEE Eng Med Biol Soc (2015) 2015:222–5. doi: 10.1109/EMBC.2015.7318340 26736240

[125] Dannhauer M Huang Z Beynel L Wood E Bukhari-Parlakturk N Peterchev AV . TAP: targeting and analysis pipeline for optimization and verification of coil placement in transcranial magnetic stimulation. J Neural Eng (2022) 19(2). doi: 10.1088/1741-2552/ac63a4 PMC9131512 35377345

[126] Kammer T Beck S Thielscher A Laubis-Herrmann U Topka H . Motor thresholds in humans: a transcranial magnetic stimulation study comparing different pulse waveforms, current directions and stimulator types. Clin Neurophysiol (2001) 112(2):250–8. doi: 10.1016/S1388-2457(00)00513-7 11165526

[127] McLaren DG Ries ML Xu G Johnson SC . A generalized form of context-dependent psychophysiological interactions (gPPI): a comparison to standard approaches. Neuroimage (2012) 61(4):1277–86. doi: 10.1016/j.neuroimage.2012.03.068 PMC3376181 22484411

[128] Friston KJ Buechel C Fink GR Morris J Rolls E Dolan RJ . Psychophysiological and modulatory interactions in neuroimaging. Neuroimage (1997) 6(3):218–29. doi: 10.1006/nimg.1997.0291 9344826

[129] Nieto-Castanon A . General linear model. In: Nieto-Castanon A , editor. Handbook of functional connectivity Magnetic Resonance Imaging methods in CONN. Boston, MA: Hilbert Press (2020). p. 63–82.

[130] Nieto-Castanon A . Cluster-level inferences. In: Nieto-Castanon A , editor. Handbook of functional connectivity Magnetic Resonance Imaging methods in CONN. Boston, MA: Hilbert Press (2020). p. 83–104.

[131] Worsley KJ Marrett S Neelin P Vandal AC Friston KJ Evans AC . A unified statistical approach for determining significant signals in images of cerebral activation. Hum Brain Mapp (1996) 4(1):58–73. doi: 10.1002/(SICI)1097-0193(1996)4:1<58::AID-HBM4>3.0.CO;2-O 20408186

[132] Chumbley J Worsley K Flandin G Friston K . Topological FDR for neuroimaging. Neuroimage (2010) 49(4):3057–64. doi: 10.1016/j.neuroimage.2009.10.090 PMC3221040 19944173

[133] Whitfield-Gabrieli S Nieto-Castanon A . Conn: a functional connectivity toolbox for correlated and anticorrelated brain networks. Brain Connect (2012) 2(3):125–41. doi: 10.1089/brain.2012.0073 22642651

[134] Nieto-Castanon A Whitfield-Gabrieli S . CONN functional connectivity toolbox: RRID SCR_009550, release 22. Boston, MA: Hilbert Press (2022).

[135] Penny WD Friston KJ Ashburner JT Kiebel SJ Nichols TE . Statistical Parametric Mapping: The analysis of functional brain images. London, UK: Elsevier (2011).

[136] Nieto-Castanon A . FMRI minimal preprocessing pipeline. In: Nieto-Castanon A , editor. Handbook of functional connectivity Magnetic Resonance Imaging methods in CONN. Boston, MA: Hilbert Press (2020). p. 3–16.

[137] Desikan RS Segonne F Fischl B Quinn BT Dickerson BC Blacker D . An automated labeling system for subdividing the human cerebral cortex on MRI scans into gyral based regions of interest. Neuroimage (2006) 31(3):968–80. doi: 10.1016/j.neuroimage.2006.01.021 16530430

[138] Nieto-Castanon A . Functional connectivity measures. In: Nieto-Castanon A , editor. Handbook of functional connectivity Magnetic Resonance Imaging methods in CONN. Boston, MA: Hilbert Press (2020). p. 26–62.

[139] Ingalhalikar M Parker WA Bloy L Roberts TP Verma R . Creating multimodal predictors using missing data: classifying and subtyping autism spectrum disorder. J Neurosci Methods (2014) 235:1–9. doi: 10.1016/j.jneumeth.2014.06.030 24983132

[140] Brondino N Fusar-Poli L Panisi C Damiani S Barale F Politi P . Pharmacological modulation of GABA function in autism spectrum disorders: A systematic review of human studies. J Autism Dev Disord (2016) 46(3):825–39. doi: 10.1007/s10803-015-2619-y 26443675

[141] Fung LK Hardan AY . Developing medications targeting glutamatergic dysfunction in autism: progress to date. CNS Drugs (2015) 29(6):453–63. doi: 10.1007/s40263-015-0252-0 PMC4515163 26104862

[142] Teng S Guo Z Peng H Xing G Chen H He B . High-frequency repetitive transcranial magnetic stimulation over the left DLPFC for major depression: Session-dependent efficacy: A meta-analysis. Eur Psychiatry (2017) 41:75–84. doi: 10.1016/j.eurpsy.2016.11.002 28049085

[143] Fitzgerald PB Hoy K McQueen S Maller JJ Herring S Segrave R . A randomized trial of rTMS targeted with MRI based neuro-navigation in treatment-resistant depression. Neuropsychopharmacology (2009) 34(5):1255–62. doi: 10.1038/npp.2008.233 19145228

[144] Sale MV Mattingley JB Zalesky A Cocchi L . Imaging human brain networks to improve the clinical efficacy of non-invasive brain stimulation. Neurosci Biobehav Rev (2015) 57:187–98. doi: 10.1016/j.neubiorev.2015.09.010 26409343

[145] Lynch G Kramar EA Babayan AH Rumbaugh G Gall CM . Differences between synaptic plasticity thresholds result in new timing rules for maximizing long-term potentiation. Neuropharmacology (2013) 64(1):27–36. doi: 10.1016/j.neuropharm.2012.07.006 22820276 PMC3445784

[146] Thomson AC Sack AT . How to design optimal accelerated rTMS protocols capable of promoting therapeutically beneficial metaplasticity. Front Neurol (2020) 11:599918. doi: 10.3389/fneur.2020.599918 33224103 PMC7674552

[147] Schiena G Maggioni E Pozzoli S Brambilla P . Transcranial magnetic stimulation in major depressive disorder: Response modulation and state dependency. J Affect Disord (2020) 266:793–801. doi: 10.1016/j.jad.2020.02.006 32217261

